# HCCS Serves as Potential Prognostic Biomarker and Therapeutic Target in Human Breast Cancer

**DOI:** 10.1155/ijbc/6717594

**Published:** 2025-12-05

**Authors:** Sm Faysal Bellah, Md Alim Hossen, S. M. Saker Billah, Md. Nur Islam

**Affiliations:** ^1^ Department of Pharmacy, Bangladesh University, Dhaka, Bangladesh, bu.edu.bd; ^2^ Bangladesh University Research Center, Bangladesh University, Dhaka, Bangladesh, bu.edu.bd; ^3^ Bioinformatics Laboratory, Department of Statistics, University of Rajshahi, Rajshahi, Bangladesh, ru.ac.bd; ^4^ Department of Chemistry, National University, Gazipur, Bangladesh, inu.ac.kr; ^5^ Department of Pharmacy, Manarat International University, Dhaka, Bangladesh, manarat.ac.bd

**Keywords:** and TCGA, breast cancer, HCCS, immune infiltration, overall survival, relapse-free survival

## Abstract

**Background:**

Breast cancer is a leading cause of cancer‐related morbidity and mortality in women worldwide. Among its subtypes, triple‐negative breast cancer (TNBC) poses the greatest therapeutic challenge due to its aggressive nature and lack of targeted treatments. Holocytochrome c synthase (HCCS), a mitochondrial enzyme essential for cytochrome c maturation, may play a pivotal role in cancer pathogenesis.

**Objective:**

This study aimed to investigate the expression profile, epigenetic regulation, immune interactions, prognostic significance, and molecular networks of HCCS across cancers, with a particular focus on breast cancer and its subtypes.

**Methods:**

Publicly available datasets and bioinformatics tools were employed to analyze HCCS expression, methylation, survival outcomes, immune infiltration, and interaction networks. Expression and clinical outcomes were examined using TCGA, while methylation and expression patterns were assessed via UALCAN and TNMplot. Survival analyses were performed using Kaplan–Meier Plotter, and immune infiltration was evaluated with TIMER2.0. Protein–protein interaction networks were generated with STRING, and functional enrichment was conducted through g:Profiler. Key findings were validated in independent breast cancer cohorts from GEO and the GOBO platform.

**Results:**

HCCS was significantly overexpressed in multiple cancers, with the highest upregulation observed in breast cancer, particularly TNBC. Hypomethylation of the HCCS promoter was associated with increased expression. High HCCS expression correlated with poorer relapse‐free survival and greater immune infiltration, including CD4^+^ T cells, CD8^+^ T cells, macrophages (M1/M2), mast cells, and regulatory T cells. Protein–protein interaction analysis revealed HCCS‐associated genes enriched in mitochondrial and apoptotic pathways. Validation across independent datasets consistently supported the association of elevated HCCS expression with poor prognosis in breast cancer.

**Conclusion:**

This integrated bioinformatics analysis highlights HCCS as a potential prognostic biomarker and therapeutic target in breast cancer, particularly in TNBC, although further experimental validation is required before clinical application.

## 1. Introduction

Breast cancer is the most commonly diagnosed cancer in women globally and remains a major cause of cancer‐related death, despite improvements in early detection and therapy [1]. In 2022, it accounted for an estimated 2.3 million new cases, representing ~ 11.6% of all cancer diagnoses globally [2]. As one of the most prevalent malignancies in women, breast cancer’s high incidence and mortality underscore the ongoing need for improved strategies in early detection, molecular profiling, and personalized treatment [3]. It is a heterogeneous disease comprising distinct molecular subtypes, each with unique biological features, clinical outcomes, and therapeutic responses. Among these, triple‐negative breast cancer (TNBC) is particularly aggressive, as it lacks estrogen receptor (ER), progesterone receptor (PR), and HER2 expression, rendering it unresponsive to endocrine or HER2‐targeted therapies [4–6]. TNBC is associated with poor prognosis, high recurrence rates, and limited treatment options, highlighting the urgent need for novel biomarkers and therapeutic targets [7]. Breast cancer pathogenesis is driven by a complex interplay of genetic factors, environmental factors (such as hormonal exposure and lifestyle influences), and abnormal cellular signaling pathways. Furthermore, various cytokines, including CCL18 and EGF, are implicated in fostering chronic inflammation, tumor progression, immune suppression, and metastasis [8]. Collectively, these elements underlie the heterogeneity and aggressive nature of distinct breast cancer subtypes [9]. Identifying novel biomarkers and therapeutic targets for TNBC is, therefore, a critical unmet need.

Mitochondria, beyond their classical role in ATP production, are central to the regulation of apoptosis, oxidative stress, and cellular metabolism—all processes implicated in tumor development and progression [10]. One mitochondrial gene of interest is holocytochrome c synthase (HCCS), an essential enzyme encoded by the nuclear genome and imported into mitochondria. HCCS catalyzes the covalent attachment of heme to apocytochrome c, forming holocytochrome c, a key player in electron transport and intrinsic apoptosis [11]. Lyase catalyzes the covalent linking of the heme group to the cytochrome C apoprotein to produce the mature functional cytochrome [12]. Dysregulation of HCCS can impair mitochondrial respiration and apoptotic signaling, enabling cancer cells to evade cell death. Recent studies suggest that altered expression of mitochondrial genes, including HCCS, may be associated with tumor aggressiveness and therapy resistance [10, 13]. Furthermore, epigenetic mechanisms such as promoter DNA methylation may regulate HCCS expression, as aberrant methylation is frequently observed in cancer and contributes to silencing of tumor suppressor genes [14].

Although HCCS has been studied in mitochondrial disorders, its role in cancer—particularly breast cancer—remains largely unexplored [15]. Given the importance of mitochondrial dynamics in tumor biology, HCCS dysregulation could influence cancer cell survival, metabolic reprogramming, and resistance to therapy. Epigenetic modifications such as DNA methylation may further drive its aberrant expression [14]. Additionally, mitochondrial proteins are increasingly recognized as modulators of immune responses in the tumor microenvironment [16], raising the possibility that HCCS may also contribute to immune evasion.

Emerging evidence suggests that HCCS plays roles beyond mitochondrial metabolism, with links to neurodevelopmental disorders, mitochondrial cytopathies, and cardiovascular diseases, underscoring its systemic biological importance [17–20]. Mutations or dysregulation of HCCS have been implicated in microphthalmia with linear skin defects (MLS) syndrome and mitochondrial encephalomyopathies, highlighting its essential role in cell survival, oxidative phosphorylation, and differentiation [20, 21]. In cancer, particularly TNBC—a subtype characterized by metabolic rewiring and defective apoptosis—aberrant expression of mitochondrial regulators such as HCCS may create a permissive environment for tumor progression and therapy resistance [22]. This suggests HCCS may serve as a molecular link between mitochondrial dysfunction, apoptosis resistance, and cancer pathobiology.

HCCS was prioritized in this study due to its central role in mitochondrial biology and emerging links to human disease. As the sole enzyme catalyzing the covalent attachment of heme to apocytochrome c, HCCS is indispensable for holocytochrome c formation, oxidative phosphorylation, and intrinsic apoptotic signalling. Despite its fundamental role, the oncogenic potential of HCCS remains poorly characterized compared to other mitochondrial regulators. Prior studies have connected HCCS mutations to rare mitochondrial syndromes such as MLS, but its role in breast cancer progression and prognosis remains unclear.

Bioinformatics has been widely applied in cancer research, particularly for the identification of novel cancer biomarkers [23–25]. By leveraging public databases, valuable insights can be gained into gene expression, mutation, and immune associations, thereby improving the efficiency and accuracy of biomarker discovery. In this context, the present study evaluates the expression patterns, epigenetic regulation, mutational landscape, and immune associations of HCCS in breast cancer using bioinformatics approaches. Through integrative analyses of publicly available transcriptomic and clinical datasets, we investigate the prognostic potential of HCCS and its relevance across breast cancer subtypes, with a particular focus on TNBC. Our findings provide new insights into the molecular functions of HCCS and highlight its potential clinical utility in cancer diagnosis, prognosis, and therapy, although further experimental validation is necessary to confirm its translational applicability.

## 2. Material and Method

### 2.1. Analysis of HCCS Expression Across Multiple Cancer Types Using TCGA Data

To investigate the expression profile of the HCCS gene across diverse tumor types, we utilized the TIMER2.0 web‐based platform (http://timer.cistrome.org/), which facilitates comprehensive analysis of gene expression and immune cell infiltration using data from The Cancer Genome Atlas (TCGA). Specifically, the “Gene_DE” module was used to assess differences in HCCS mRNA expression between primary tumor samples and their corresponding normal tissues across various cancer types, which processes log2‐transformed transcripts per million (TPM) values for normalization, ensuring comparability across samples. TIMER2.0 integrates standardized RNA sequencing data from TCGA, providing robust tools for differential expression analysis and enabling visualization of expression trends across a broad spectrum of malignancies. This approach supports the systematic evaluation of HCCS gene regulation within the context of tumor biology and the tumor microenvironment [26]. Multiple publicly available databases were utilized to further evaluate HCCS expression across various cancer types and tissue origins. The Cancer section of the Human Protein Atlas (HPA) (https://www.proteinatlas.org) was accessed to examine both mRNA and protein expression patterns, supported by immunohistochemical imaging data across a wide range of tumor tissues. The HPA database reports mRNA expression levels in protein‐coding transcripts per million (pTPM). The pTPM normalization specifically scales expression values based on the total number of protein‐coding transcripts rather than all RNA species, ensuring more accurate comparisons of protein‐related gene expression across samples. For RNA‐seq‐based comparative analysis, TNMplot (https://tnmplot.com/analysis/) was employed to assess HCCS expression across normal, tumor, and metastatic tissues using its pan‐cancer visualization and gene signature tools [27]. Additionally, gene expression data from the Genotype‐Tissue Expression (GTEx) project (https://gtexportal.org) were used to compare HCCS transcript levels in healthy tissues [28]. GTEx expression values, reported in TPM, were generated using RNA‐SeQC after rigorous filtering, with gene annotations refined to remove intronic and overlapping transcript artifacts [29]. The expression level of TPM was then computed as log2‐transcript per million (Log2‐TPM).

### 2.2. Analysis of HCCS Expression in Breast Cancer

To specifically analyze HCCS expression in breast cancer, the UALCAN online platform (http://ualcan.path.uab.edu) was utilized. This tool provides access to TCGA transcriptomic data and enables subgroup‐specific analysis of gene expression based on clinicopathological parameters. Expression levels of HCCS were visualized through box‐and‐whisker plots, allowing comparisons between tumor subtypes and normal breast tissue samples. The database reports mRNA expression levels in transcripts per million (TPM). The expression level of TPM was then computed as log2‐transcript per million (Log2‐TPM). This approach facilitated the identification of expression patterns associated with breast cancer progression and molecular subtypes [30, 31].

### 2.3. Assessment of Promoter DNA Methylation Profile of HCCS in Breast Cancer

To investigate potential epigenetic mechanisms regulating HCCS expression in breast cancer, promoter DNA methylation levels were assessed using the UALCAN web resource (http://ualcan.path.uab.edu). This platform incorporates TCGA‐derived methylation data to facilitate the evaluation of gene‐specific promoter methylation across cancer types. For this study, breast cancer datasets were selected to examine methylation patterns at the HCCS promoter region. Methylation intensity was quantified using beta values, which range from 0 (indicating no methylation) to 1 (indicating complete methylation). This analysis was conducted to explore whether changes in promoter methylation could be linked to dysregulated HCCS expression in breast cancer tissues [30].

### 2.4. Analysis of HCCS Mutations and Copy Number Variations

To characterize the mutational landscape of HCCS across human cancers, data were retrieved from the Catalogue of Somatic Mutations in Cancer (COSMIC, GRCh38, version 97). COSMIC provides curated information on somatic mutations identified in a wide range of tumor types. The types and frequencies of HCCS mutations were analyzed, and their distribution was visualized using pie charts to illustrate the relative proportions of different mutation classes. In addition, copy number variation (CNV) data were assessed to identify potential genomic alterations that may contribute to aberrant HCCS expression or function in cancer [32, 33].

### 2.5. Survival Analysis Based on HCCS Expression in Breast Cancer

To assess the prognostic relevance of HCCS expression in breast cancer, survival analysis was performed using the Kaplan–Meier Plotter (http://www.kmplot.com), an online resource that integrates gene expression profiles with clinical outcome data. The prognostic significance of HCCS mRNA expression in breast cancer was evaluated [31, 34]. This dataset contained relapse‐free survival (RFS) information for 1336 patients, distant metastasis‐free survival (DMFS) data for 2765 patients, and overall survival (OS) data for 1061 individuals. Patient cohorts were stratified into high and low expression groups based on the median HCCS mRNA expression level. Survival outcomes, including RFS and OS, were evaluated using Kaplan–Meier curves, with the number of patients at risk displayed below each plot. The analysis yielded hazard ratios (HR), 95% confidence intervals, and log‐rank *P*‐values, with statistical significance defined as *p* < 0.05. To ensure data quality, arrays failing quality control checks were excluded from the analysis. Additionally, survival data from TCGA were analyzed through the UALCAN platform (http://ualcan.path.uab.edu) to validate findings and explore associations between HCCS expression and survival probability, with subgroup analysis based on menopausal status [30, 31].

### 2.6. Gene Correlation Analyses of HCCS Expression

To explore potential functional partners and co‐expressed genes associated with HCCS, transcriptomic correlation analysis was performed using the LinkedOmics platform (https://www.linkedomics.org). This tool allows for large‐scale correlation studies based on TCGA RNA‐sequencing data. Pearson correlation coefficients were calculated between HCCS and all other protein‐coding genes across the dataset. Genes showing statistically significant positive or negative correlations with HCCS expression were identified for further interpretation. To enhance data reliability and reduce noise, genes with extremely low expression (Z score < −4) were excluded from the analysis [35].

### 2.7. Construction of Protein–Protein Interaction Network for HCCS

To investigate the functional protein interaction landscape of HCCS, a protein–protein interaction (PPI) network was generated using the STRING database (http://string-db.org) [36]. This resource integrates both experimentally confirmed and computationally predicted interactions to provide a comprehensive view of protein connectivity. The analysis was restricted to Homo sapiens, and a high‐confidence interaction score threshold of 0.9 was applied to ensure robustness and minimize false positives. This network analysis enabled the identification of potential protein partners of HCCS, offering insights into its involvement in key biological pathways and molecular functions [37].

### 2.8. Gene Set Enrichment Analysis

To elucidate the biological processes, molecular functions, and pathways associated with HCCS and its correlated genes in breast cancer, we performed a comprehensive Gene Set Enrichment Analysis (GSEA). The analysis was conducted using the g:Profiler web server (https://biit.cs.ut.ee/gprofiler/gost), which provides access to updated Gene Ontology (GO) annotations and KEGG pathway databases [38]. The input gene set consisted of HCCS and its 10 significantly correlated genes identified in our study: HCCS, COX10, FECH, ARHGAP6, HMOX2, HMOX1, COX15, CYCS, CYC1, gastrin‐releasing peptide receptor (GRPR), and TIMMDC1. The g:GOSt tool within g:Profiler was used to identify over‐represented GO terms in the categories of Biological Process (BP), Molecular Function (MF), Cellular Component (CC), and KEGG pathways. Statistical significance was determined using the hypergeometric test, with a significance threshold of a corrected *p* value (*p*_adj_) < 0.05 after applying the Benjamini–Hochberg false discovery rate (FDR) procedure for multiple testing correction [39]. The Ensembl database served as the underlying genomic annotation source [40].

### 2.9. Validation of HCCS Expression in Independent Datasets

To further confirm the expression pattern of HCCS, we evaluated the GSE65194 dataset from the Gene Expression Omnibus (GEO) database. This dataset includes 167 breast tumor and 11 normal breast tissue samples, generated using the Affymetrix Human Genome U133 Plus 2.0 array. After preprocessing, the expression levels of HCCS were extracted and statistically compared between tumor and normal tissues using a two‐tailed Student’s *t* test. We also explored the expression of HCCS across molecular subtypes of breast cancer using the GOBO (Gene expression‐based Outcome for Breast cancer Online) platform (http://co.bmc.lu.se/gobo). GOBO integrates data from 1881 breast cancer cases, compiled from 11 public datasets based on Affymetrix array profiling [41]. HCCS transcript levels were analyzed across molecular subtypes and histological grades using one‐way analysis of variance (ANOVA), while associations with estrogen receptor (ER) status were assessed using the Student’s *t* test. A threshold of *p* < 0.05 was considered statistically significant for all analyses.

### 2.10. Validation of the Prognostic Value of HCCS in Breast Cancer

The prognostic relevance of HCCS expression was further assessed using the GOBO outcome analysis module. Survival analyses were performed for OS, DMFS, and RFS with a 10‐year censoring limit. Patients were divided into three groups (low, medium, and high) according to HCCS expression tertiles. A Cox proportional hazards regression model was applied to evaluate the association between HCCS expression and survival outcomes, adjusting for ER status, nodal status, tumor grade, age, and tumor size. Log‐rank *p*‐values were transformed into –log10 (*p*) values for visualization. Associations with *p* < 0.05 were considered statistically significant.

## 3. Statistical Analysis

Statistical significance was determined using a standardized threshold, with results considered significant at *p* < 0.05. For expression, the expression level of TPM was computed as log2‐transcript per million (Log2‐TPM). In survival analyses, log‐rank (*p* value) tests were employed to compare survival distributions between groups, and hazard ratios (HRs) were calculated along with their 95% confidence intervals to estimate risk. The significance of findings was annotated as follows: ns for nonsignificant results, ∗ for *p* < 0.05, ∗∗ for *p* < 0.01, and ∗∗∗ for *p* < 0.001.

## 4. Results

### 4.1. Expression Profile of HCCS in Normal Human Tissues and TCGA Cancer Types

To establish a baseline expression pattern of HCCS, we first examined its mRNA and protein levels across a range of normal human tissues using data from the Genotype‐Tissue Expression (GTEx) project. The analysis revealed that HCCS exhibits a tissue‐specific expression profile, suggesting its involvement in distinct physiological processes (Figure S1).

To assess the dysregulation of HCCS in cancer, we employed RNA‐sequencing datasets from TCGA using the TIMER2.0 platform, which compares gene expression between tumor and matched normal tissues. HCCS was found to be significantly upregulated in multiple cancer types, including breast invasive carcinoma (BRCA), kidney chromophobe (KICH), lung adenocarcinoma (LUAD), lung squamous cell carcinoma (LUSC), and uterine corpus endometrial carcinoma (UCEC) (Figure 1a). These findings suggest a potential tumor‐promoting role for HCCS in certain cancer types; however, the magnitude and significance of expression changes varied across cancers, reflecting a context‐dependent regulatory mechanism. We further validated these expression patterns using the RNA‐Seq data from normal and cancer tissues in TNMplot. This analysis confirmed elevated HCCS expression in various malignancies, notably in cancers of the adrenal gland, bladder, breast, esophagus, liver, lung, ovary, pancreas, prostate, rectum, kidney, skin, stomach, testis, thyroid, and uterus (Figure 1b). These results were also supported by additional pan‐cancer RNA‐seq comparisons from TCGA samples (Figure 1c), reinforcing the observation that HCCS is overexpressed in several tumor types compared to normal tissues.

Figure 1Differential expression of HCCS across multiple cancer types using TCGA data. (a) HCCS expression across various cancer types was analyzed using the TIMER2.0 platform, which enables comparison of gene expression between tumor and adjacent normal tissues from TCGA datasets. Box plots display expression distributions, and statistical differences were evaluated using the Wilcoxon test. Levels of significance are indicated as follows: ∗*p* < 0.05, ∗∗*p* < 0.01, and ∗∗∗*p* < 0.001. Gray columns represent cancer types with available adjacent normal tissue data, highlighting genes with significant up‐ or down‐regulation in tumors. (b) The TNMplot tool was used to further assess HCCS expression in tumor versus normal tissues (https://tnmplot.com/analysis/). Only samples with expression values greater than 10 were included in the analysis. Statistically significant differences were determined using the Mann–Whitney *U* test and are marked with a red asterisk (∗*p* < 0.05). (c) Pan‐cancer analysis of HCCS gene expression was performed using the UALCAN database (https://ualcan.path.uab.edu/cgi-bin/Pan-cancer.pl?genenam=HCCS). Boxplots display HCCS expression across different tumor types, with red boxes indicating expression levels in tumor tissues and blue boxes representing corresponding normal tissues, providing insight into gene dysregulation across malignancies.

**Figure:**
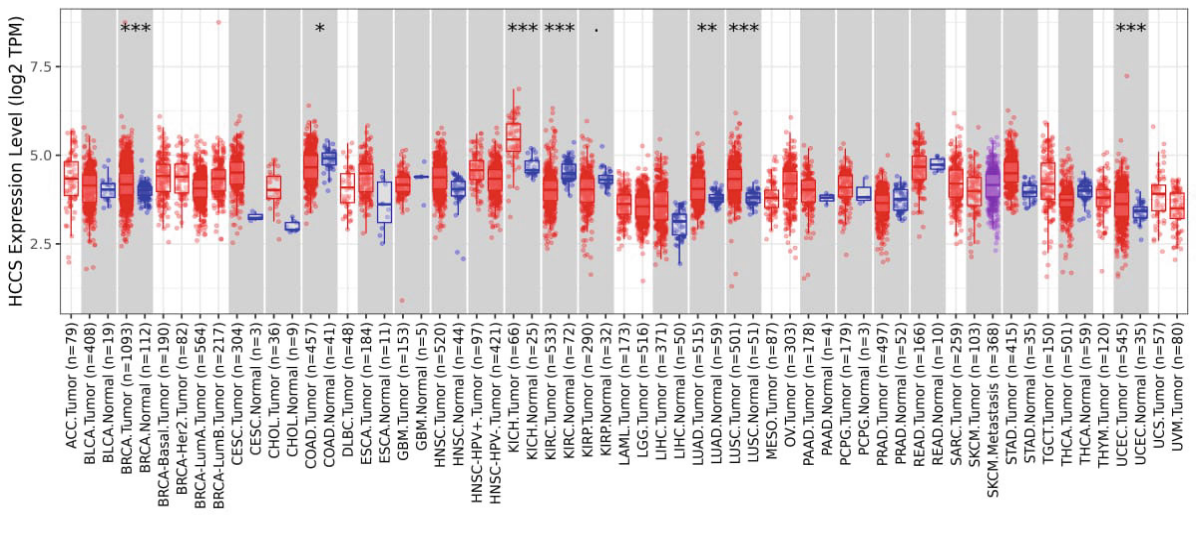
(a)

**Figure:**
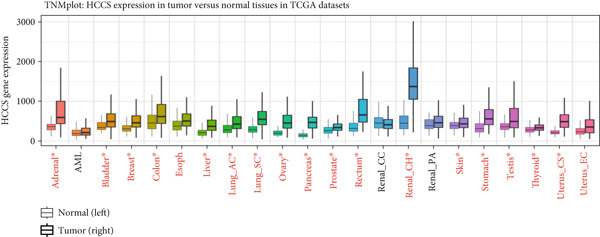
(b)

**Figure:**
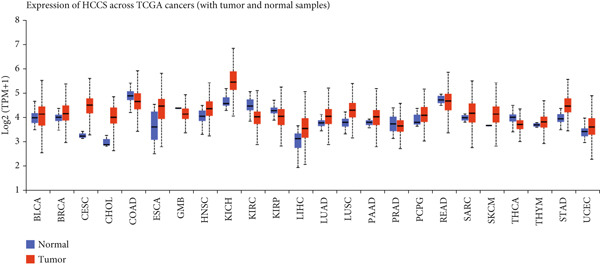
(c)

To strengthen these observations, we examined HCCS expression at both the mRNA and protein levels in tumor tissues. The transcriptomic analysis supported high expression levels of HCCS across several tumor types (Figure 2a,b), consistent with proteomic data. Given the observed differential expression, we next focused specifically on breast cancer to determine the clinical relevance of HCCS. Analysis of TCGA breast cancer datasets revealed significantly elevated HCCS expression in both primary and metastatic breast cancer when compared to normal breast tissues (Figure 2c,d).

Figure 2Overview of HCCS expression in TCGA cancer data sets. (a) RNA expression levels of HCCS across different cancer types were analyzed using the Protein Atlas database (https://www.proteinatlas.org/ENSG00000004961-HCCS/cancer), highlighting its cancer specificity. Expression data were obtained from a variety of TCGA cancer datasets, showing the variation of HCCS expression levels across different tumor types. The expression level were computed as log2‐transcript per million (Log2‐TPM). (b) Protein expression of HCCS in various TCGA cancer datasets was also assessed through the Protein Atlas (https://www.proteinatlas.org/ENSG00000004961-HCCS/cancer), providing a comprehensive overview of protein‐level expression across different cancers with an emphasis on low cancer specificity. (c) HCCS gene signature expression in breast adenocarcinoma was analyzed using RNA‐seq data from TNM plot (https://tnmplot.com/analysis/). The normal, tumor, and metastatic analysis page provides an in‐depth RNA‐seq–based assessment of a chosen gene across a specified tissue type. Statistical significance was determined using Kruskall–Wallis test, and the *p* values were calculated as compared to that of normal. The significance levels indicated as follows: ns: not significant, ∗*p* < 0.05, ∗∗*p* < 0.01, and ∗∗∗*p* < 0.001. (d) HCCS gene expression in breast adenocarcinoma accounting the normal, tumor, and metastatic using RNA‐seq based data (https://tnmplot.com/analysis/). Statistical significance was determined using the Dunnet test, and the *p* values were calculated as compared to that of normal. The significance levels indicated as follows: ns: not significant, ∗*p* < 0.05, ∗∗*p* < 0.01, and ∗∗∗*p* < 0.001.

**Figure:**
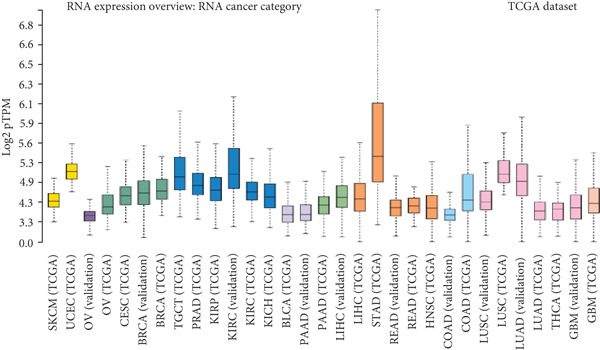
(a)

**Figure:**
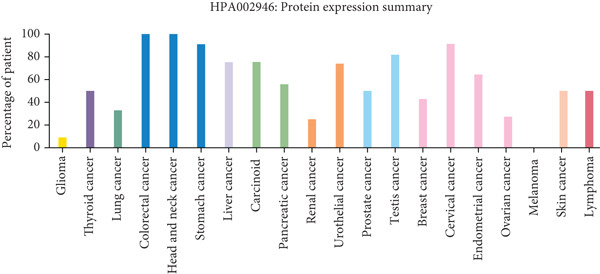
(b)

**Figure:**
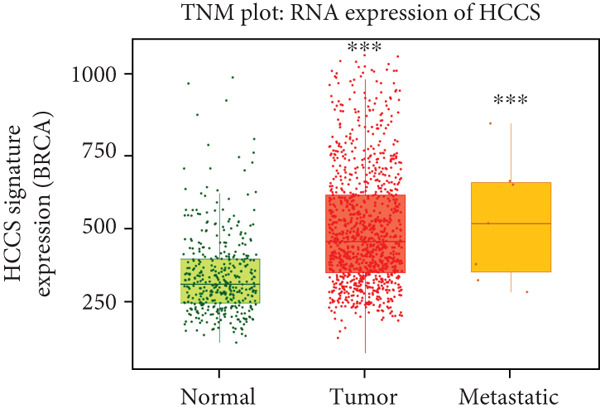
(c)

**Figure:**
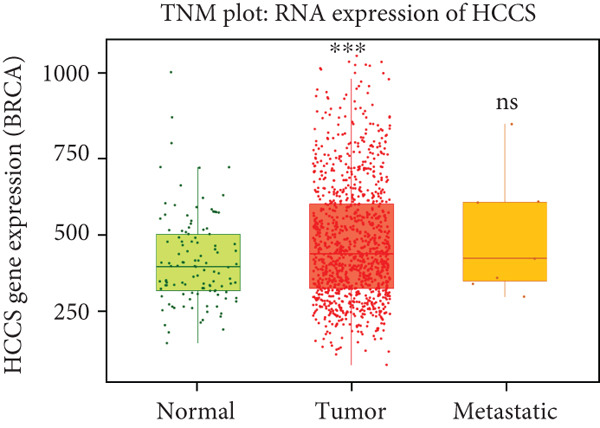
(d)

To determine whether HCCS dysregulation was unique to breast cancer or part of a broader oncogenic pattern, we extended our analysis to other tumor types not previously highlighted in TCGA pan‐cancer analysis. Using TNMplot, we analyzed the expression of HCCS in several cancers, including cervical squamous cell carcinoma (CESC), head and neck squamous cell carcinoma (HNSC), thyroid carcinoma (THCA), and pheochromocytoma and paraganglioma (PCPG). Importantly, HCCS expression did not significantly differ between tumor and normal tissues (Figures S2A–D), reinforcing the notion that its dysregulation is not universal across malignancies.

These findings suggest a potential oncogenic role for HCCS in breast cancer development and progression, warranting further investigation into its functional impact and underlying regulatory mechanisms.

### 4.2. Expression and Clinical Relevance of HCCS in Breast Cancer

Following our pan‐cancer findings, we focused our analysis on breast cancer to explore the clinical significance of HCCS expression in greater detail. Using TCGA‐BRCA transcriptomic data accessed via UALCAN, we confirmed that HCCS expression is significantly upregulated in breast tumor tissues compared to normal breast tissues (Figure 3a). This differential expression suggests a potential oncogenic role for HCCS in breast tumor biology.

Figure 3Expression of HCCS in breast adenocarcinoma (BRCA). Boxplots illustrate the expression levels of HCCS in BRCA stratified by various clinical and demographic factors. (a) Expression distribution according to patient sample type (e.g., tumor vs. normal tissues). (b) Expression relative to nodal metastasis stages. (c) Comparative expression based on patient gender. (d) Stratification by race. (e) Expression levels across individual cancer stages (stages 1–4). (f) Comparison based on menopause status. (g) Age‐based analysis of HCCS expression. (h) Subtype‐specific expression patterns across BRCA subclasses. (i) Evaluation of expression among major subgroups, including triple‐negative breast cancer (TNBC) subtypes include TNBC‐BL1 (basal‐like 1), TNBC‐BL2 (basal‐like 2), TNBC‐IM (immunomodulatory), TNBC‐M (mesenchymal), TNBC‐MSL (mesenchymal stem‐like), TNBC‐LAR (luminal androgen receptor), and TNBC‐UNS (unspecified). (j) Expression profiles based on histological classifications such as infiltrating ductal carcinoma (IDC), infiltrating lobular carcinoma (ILC), mixed (mixed histology), mucinous (mucinous carcinoma), metaplastic carcinomas, INOS (infiltrating carcinoma not otherwise specified), and medullary (medullary carcinoma). (k) Expression of HCCS in relation to TP53 mutation status. The expression level in Log2‐transcript per million (Log2‐TPM) was computed. Statistical significance was determined using appropriate tests and is indicated as follows: ns (not significant); ∗*p* < 0.05; ∗∗*p* < 0.01; ∗∗∗*p* < 0.001.

**Figure:**
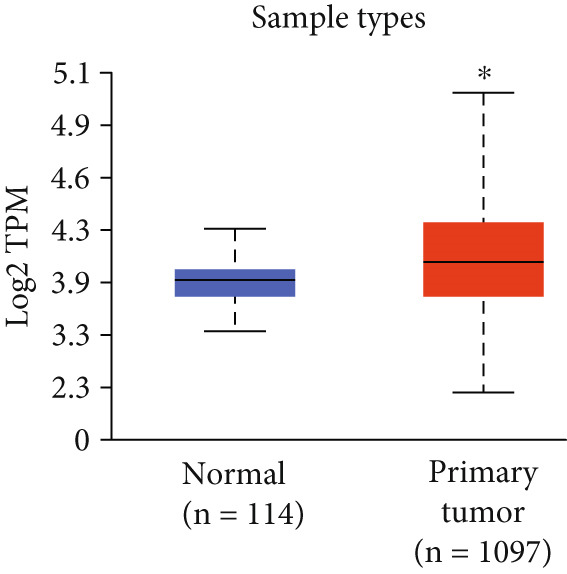
(a)

**Figure:**
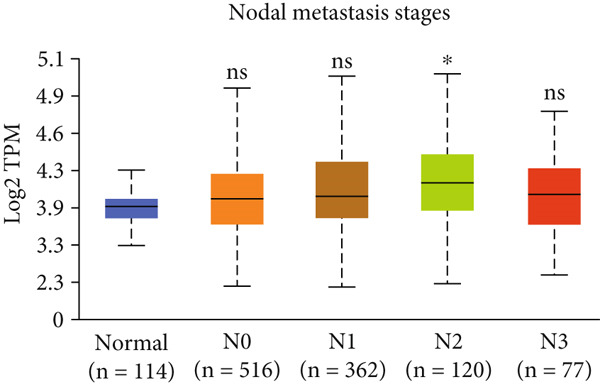
(b)

**Figure:**
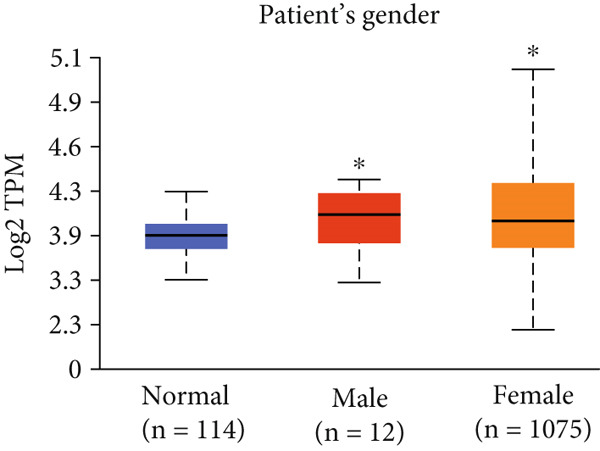
(c)

**Figure:**
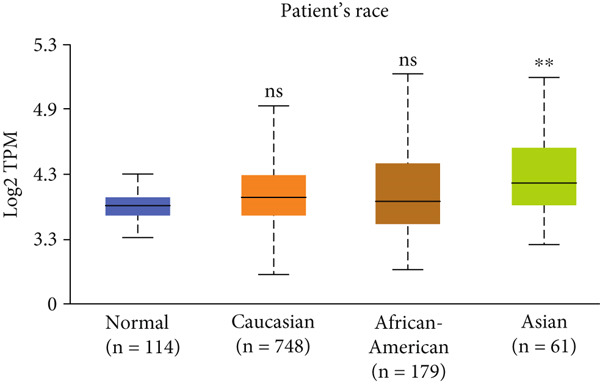
(d)

**Figure:**
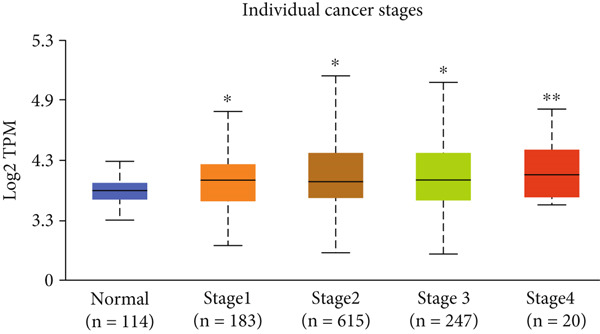
(e)

**Figure:**
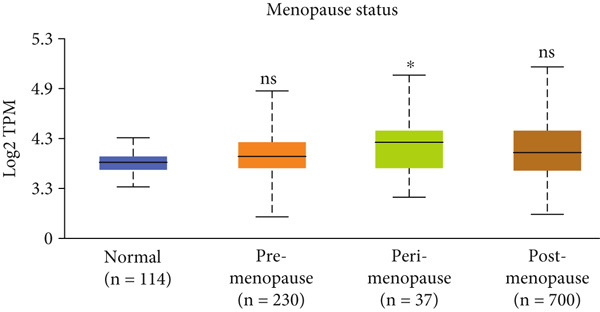
(f)

**Figure:**
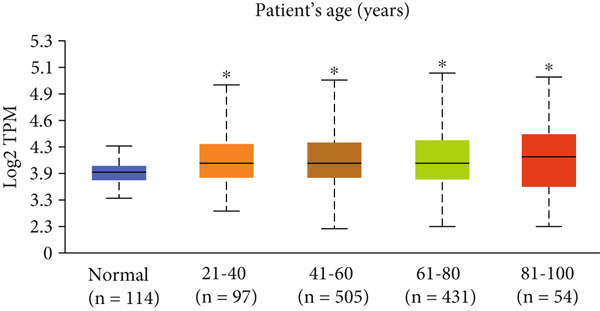
(g)

**Figure:**
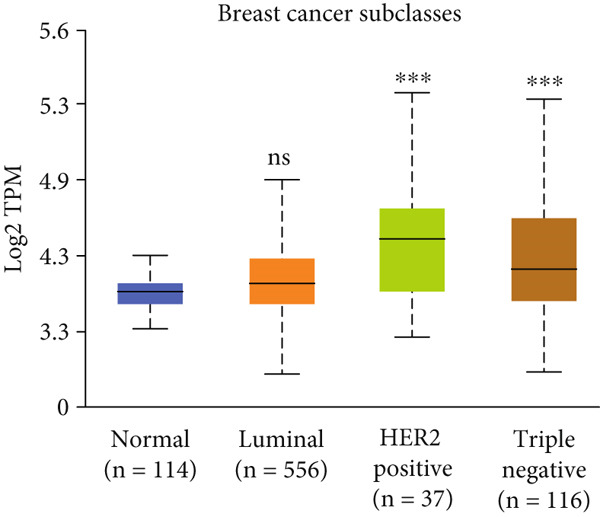
(h)

**Figure:**
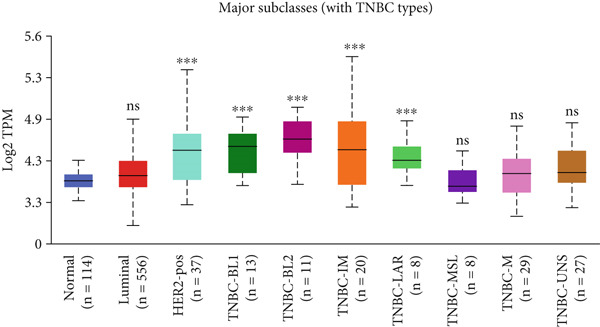
(i)

**Figure:**
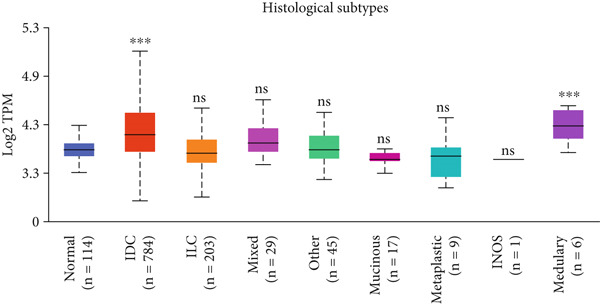
(j)

**Figure:**
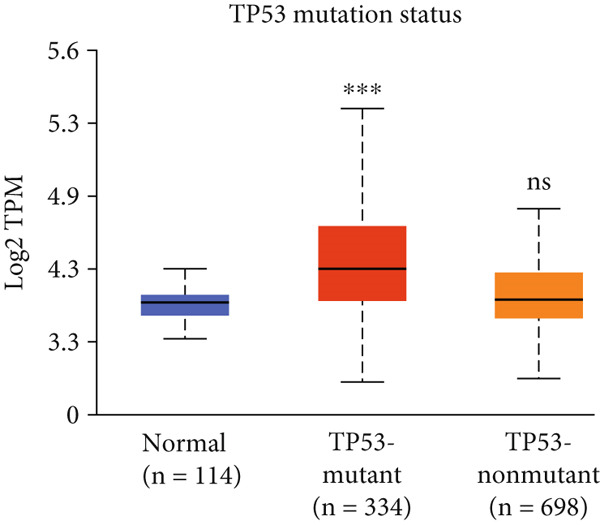
(k)

To evaluate whether HCCS expression is associated with key clinical variables, we performed a subgroup meta‐analysis stratified by clinical features, including nodal metastasis status, individual cancer stages, gender, race, menopausal status, and age (Figures 3b, 3c, 3d, 3e, 3f, and 3g). Notably, HCCS expression was significantly higher in patients with advanced nodal metastasis and later tumor stages, indicating a possible association with disease progression. However, differences observed across demographic variables (e.g., gender, race, and menopausal status) appeared less pronounced and may reflect underlying population heterogeneity rather than biological significance. These results underscore the importance of further validation in larger and more diverse cohorts.

A subtype‐specific analysis revealed that HCCS expression was most elevated in TNBC compared to other molecular subtypes, including luminal A, luminal B, and HER2‐positive tumors (Figure 3h,i). This is particularly relevant, as TNBC is associated with poor prognosis, limited therapeutic options, and aggressive clinical behavior. The elevated expression of HCCS in TNBC suggests that it may contribute to the aggressive phenotype observed in this subtype. However, we recognize that while this association is statistically significant, further functional validation and independent dataset replication are essential to substantiate its role as a TNBC‐specific biomarker. We further analyzed HCCS expression across histological subtypes of breast cancer (Figure 3j). HCCS levels were notably elevated in invasive ductal carcinoma (IDC) and medullary carcinoma compared to invasive lobular carcinoma (ILC) and other less common subtypes (Figure 3j). Given that IDC is the most prevalent and often more aggressive histological form, this observation supports the hypothesis that HCCS overexpression may be associated with more invasive breast cancer phenotypes.

Although direct tumor grade data were not analyzed, HCCS upregulation in TNBC and metastatic cases—both often correlated with higher tumor grade—implies a possible link between HCCS expression and tumor aggressiveness. Future studies, including histopathological grading and genomic instability metrics, are warranted to explore this potential association more rigorously.

In addition, we examined TP53 mutational status, given its frequent alteration in breast cancer, particularly TNBC. As TP53 mutations are often associated with defective apoptosis and metabolic reprogramming—two processes closely tied to mitochondrial function—we sought to determine whether HCCS expression correlates with TP53 mutation status. Our preliminary data suggest that HCCS expression is significantly elevated in TP53‐mutated breast cancers, raising the possibility of a functional link between p53 dysregulation and mitochondrial pathway activation involving HCCS (Figure 3k). However, mechanistic studies are needed to delineate whether this relationship is causal or merely correlative.

Overall, our findings indicate that HCCS is not only overexpressed in breast cancer but its expression correlates with aggressive clinical and molecular features, particularly in TNBC. These observations justify further investigation into the functional role of HCCS in breast cancer pathogenesis and its potential utility as a diagnostic or therapeutic target.

### 4.3. Promoter DNA Methylation of HCCS in Breast Cancer and Its Clinical Associations

Epigenetic regulation, particularly DNA methylation, plays a pivotal role in modulating gene expression and cellular functions in both normal and cancerous tissues. Specifically, promoter region hypermethylation has been implicated in the silencing of tumor suppressor genes, contributing to the aberrant gene expression profiles observed in cancers. To explore the potential epigenetic regulation of HCCS in breast cancer, we assessed the methylation status of its promoter region across various clinical and pathological features using TCGA breast cancer datasets. Methylation levels were quantified using beta values, with a range from 0 (unmethylated) to 1 (fully methylated).

Our analysis revealed that HCCS promoter methylation was significantly reduced in breast cancer tissues when compared to normal breast tissues (Figure 4a). This hypomethylation of the HCCS promoter suggests a potential mechanism by which HCCS expression may be upregulated in tumorigenic processes. Furthermore, methylation levels of HCCS showed significant variation across various patient characteristics, including sample type, nodal metastasis stage, gender, race, cancer stage, body weight, age, histological subtype, and TP53 mutation status (Figure 4).

Figure 4Promoter DNA methylation status of HCCS in BRCA. Boxplots display the promoter DNA methylation levels of the HCCS gene in BRCA samples, analyzed across various clinical and demographic categories. (a) Methylation patterns based on sample types (e.g., tumor vs. normal tissues). (b) Promoter methylation distribution according to nodal metastasis stages. (c) Methylation differences stratified by patient gender. (d) Comparison across different racial groups. (e) Promoter methylation levels across breast cancer stages (stages I–IV). (f) Analysis based on menopause status. (g) Age‐stratified methylation levels. (h) Evaluation across molecular subtypes of breast cancer. (i) Distribution of HCCS promoter methylation among different histological classifications, including infiltrating ductal carcinoma (IDC), infiltrating lobular carcinoma (ILC), mixed (mixed histology), mucinous (mucinous carcinoma), metaplastic carcinomas, INOS (infiltrating carcinoma not otherwise specified), and medullary (medullary carcinoma). (j) Analysis based on TP53 mutation status. Methylation data were obtained using the UALCAN web platform. Statistical differences were assessed using appropriate tests, and significance is indicated as follows: ns (not significant); ∗*p* < 0.05; ∗∗*p* < 0.01; ∗∗∗*p* < 0.001.

**Figure:**
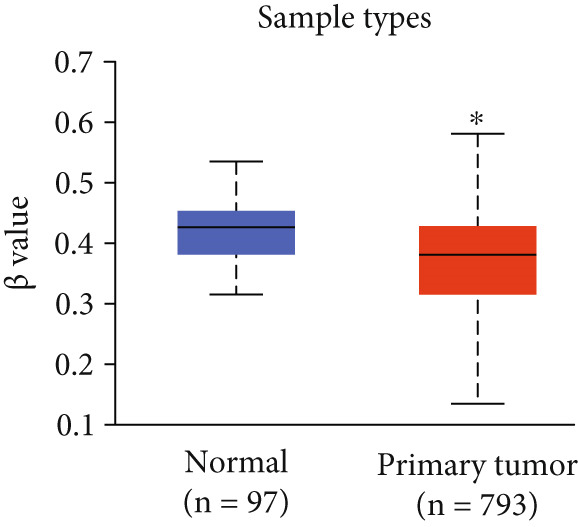
(a)

**Figure:**
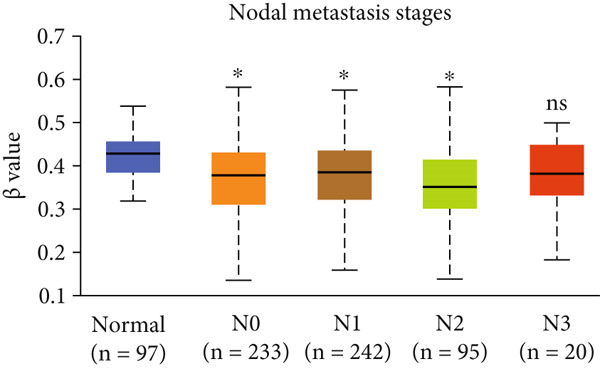
(b)

**Figure:**
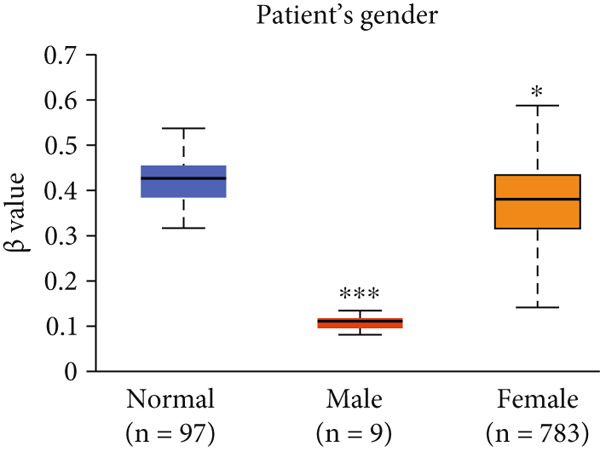
(c)

**Figure:**
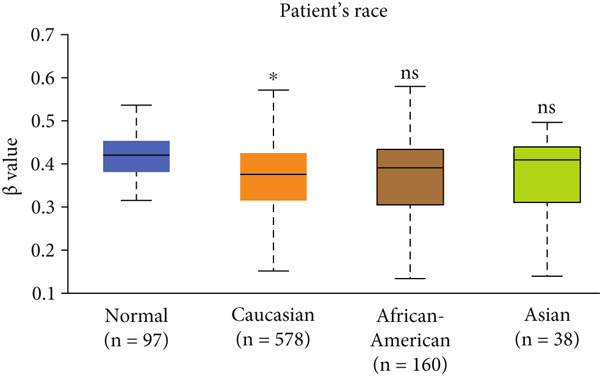
(d)

**Figure:**
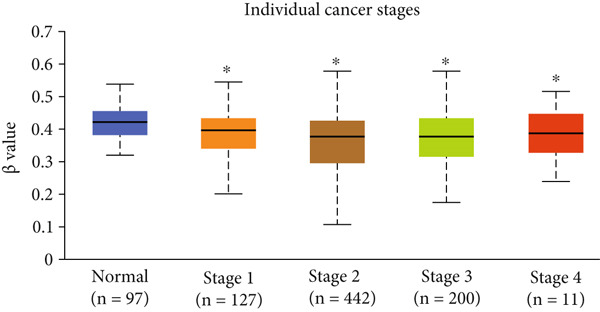
(e)

**Figure:**
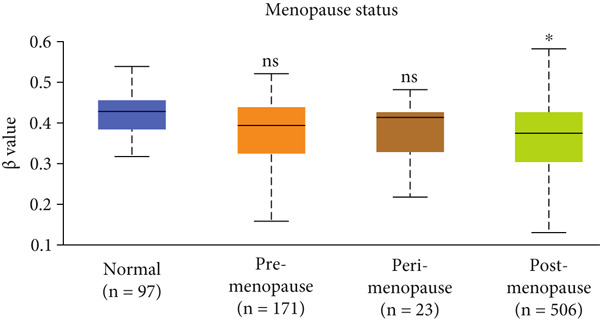
(f)

**Figure:**
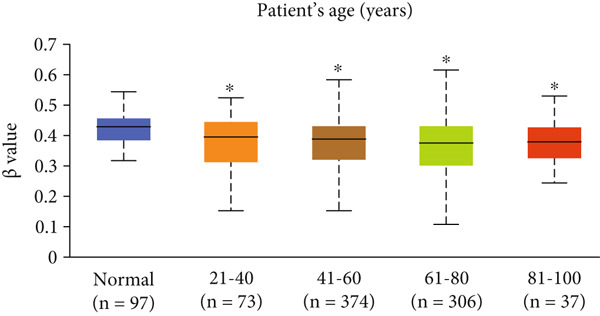
(g)

**Figure:**
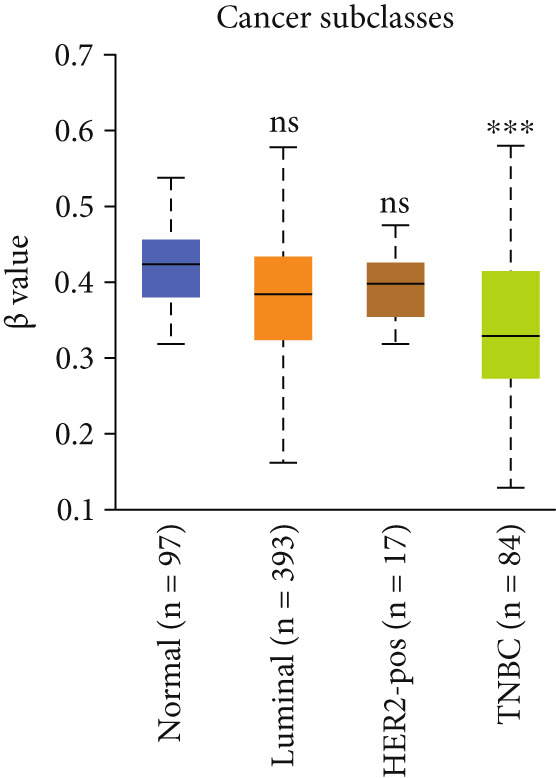
(h)

**Figure:**
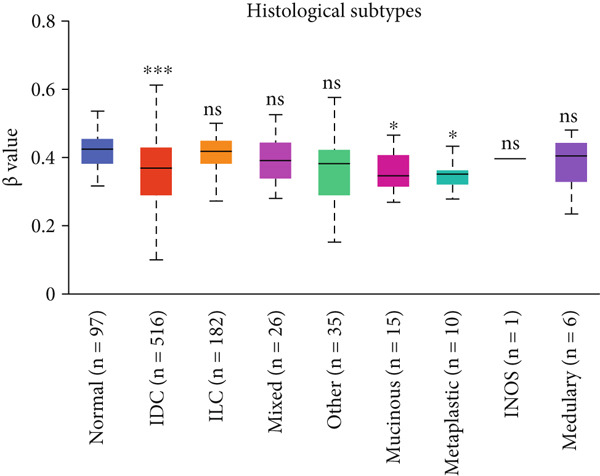
(i)

**Figure:**
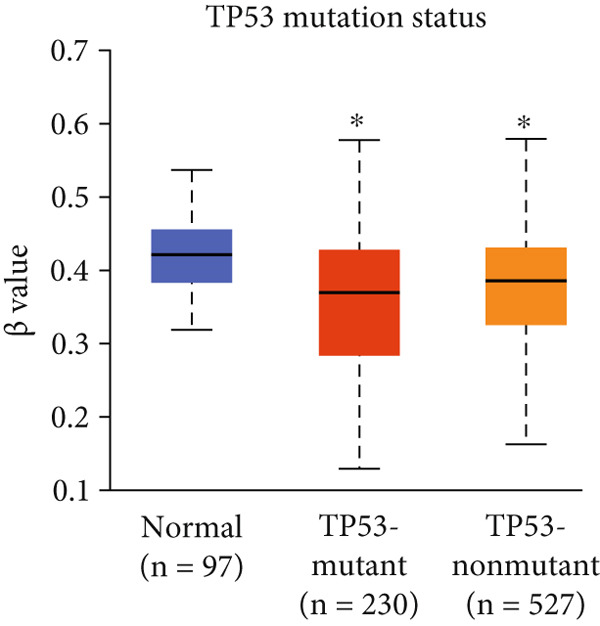
(j)

Specifically, HCCS methylation levels were significantly higher in normal breast tissues compared to primary tumors (*p* < 0.05) (Figure 4a). In addition, a trend of decreased methylation was observed as the nodal metastasis stage advanced, with lower methylation levels in stages N1, N2, and N3 (Figure 4b). Gender and race‐based differences in HCCS methylation were also observed, with lower methylation levels noted in male and Caucasian patients (Figure 4c,d).

Further analysis revealed a significant association between HCCS methylation and clinical parameters such as cancer stage (Figure 4e), menopause status (Figure 4f), patient age (Figure 4g), and breast cancer subtypes (Figure 4h). Notably, lower methylation was observed in adenocarcinoma histological subtypes compared to normal tissues (Figure 4i). TP53 mutations were also associated with lower HCCS methylation levels (Figure 4j), further linking epigenetic modifications to genetic alterations in breast cancer. These findings indicate that promoter hypomethylation may contribute to the up‐regulation of HCCS expression in breast cancer, which could play a role in tumor initiation and progression.

### 4.4. Mutational Landscape of HCCS in Cancer

To further investigate the genetic alterations of HCCS that may contribute to its dysregulation in cancer, we explored its mutational landscape using data from the Catalogue of Somatic Mutations in Cancer (COSMIC) database. Mutational profiling is crucial for understanding how genetic changes influence cancer development. Our analysis identified two primary types of mutations in HCCS: missense mutations, which alter amino acid sequences and may disrupt protein function, and synonymous mutations, which, while not changing the protein sequence, may influence gene regulation, mRNA stability, or splicing efficiency (Figure 5a).

Figure 5HCCS mutations across all primary tissue types. Pie charts illustrate (a) the distribution of HCCS mutations and (b) the proportion of different substitution mutation types across all cancer types in the TCGA cohort. The analysis was performed using mutational data obtained from the COSMIC database (https://cancer.sanger.ac.uk/cosmic/gene/analysis?ln=HCCS#distribution).

**Figure:**
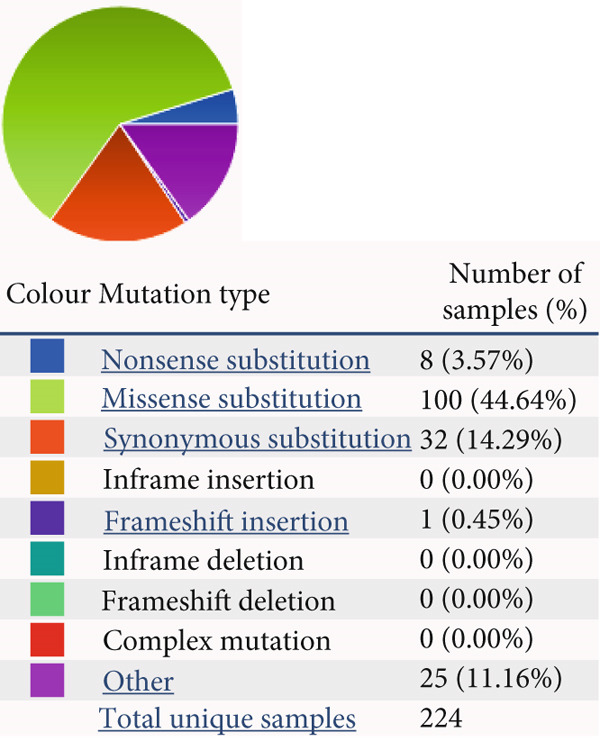
(a)

**Figure:**
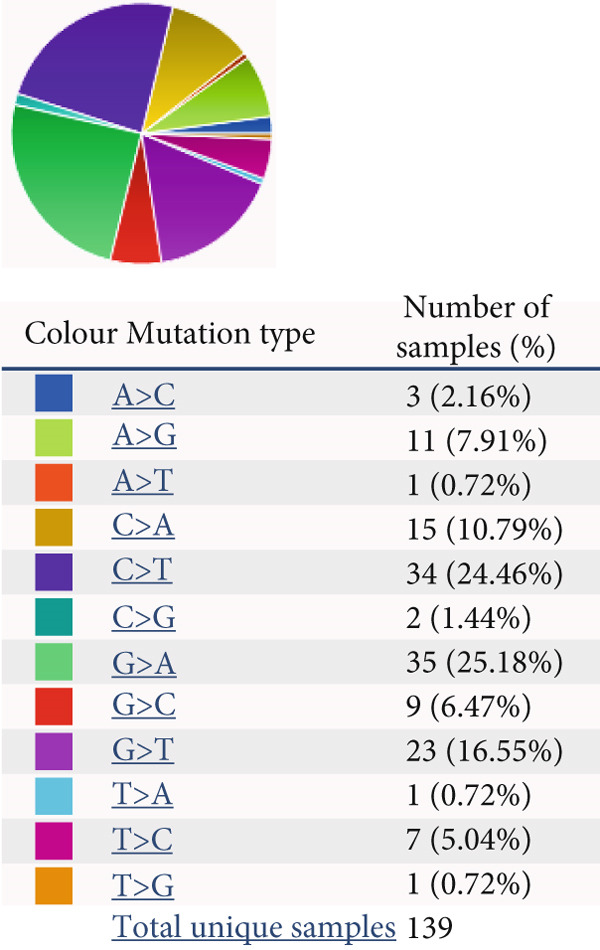
(b)

A more detailed breakdown of the mutations revealed that nucleotide substitutions, such as A>G, C>A, C>T, G>A, G>C, and G>T, were distributed across the HCCS coding region, accounting for 7.91%, 10.79%, 24.46%, 25.18%, 6.47%, and 16.55%, respectively (Figure 5b). These mutations may impact mRNA processing, translation, or protein stability, contributing to the dysregulation of HCCS in cancer cells. Importantly, the distribution of HCCS mutations across multiple cancer types—including breast, lung, prostate, ovarian, liver, skin, testicular, and penile cancers—suggests that these genetic alterations could have tissue‐specific oncogenic effects.

Given the significant relationship between HCCS expression, its methylation status, and cancer progression, these mutational alterations further highlight HCCS’s potential role in tumorigenesis. Its mutational spectrum and involvement in regulatory pathways suggest it may function as an important modulator of cancer development and progression.

### 4.5. Clinical Outcome of HCCS Expression in Breast Cancer

To further elucidate the clinical implications of HCCS expression in breast cancer prognosis, comprehensive survival analyses were conducted using data from TCGA. Utilizing the Kaplan–Meier Plotter (http://www.kmplot.com) and the Affymetrix probe ID 203745_at, we evaluated OS, (RFS, and DMFS in relation to HCCS mRNA expression levels. Patients were stratified into high‐ and low‐expression groups based on median HCCS expression levels.

In the OS analysis, while a trend towards poorer survival was observed in patients with elevated HCCS expression, the association did not reach statistical significance (*n* = 1879, HR = 0.9 [0.9–1.31], *p* = 0.4) (Figure 6a). Notably, patients with lower HCCS expression exhibited a marginally better OS outcome than those with higher expression, suggesting that lower HCCS expression may be associated with a favorable prognosis in breast cancer. However, this finding warrants further validation with larger patient cohorts.

Figure 6Prognostic impact of HCCS expression on survival outcomes in breast cancer patients. Kaplan–Meier and subgroup survival analyses were conducted to evaluate the association between HCCS expression levels and clinical outcomes in BRCA (a, b, and c). (a) Kaplan–Meier survival curve displaying overall survival (OS) among 1061 BRCA patients stratified by HCCS expression levels. (b) Relapse‐free survival (RFS) curve based on HCCS expression among 1336 BRCA patients. (c) Distant metastasis‐free survival (DMFS) curve based on HCCS expression among 2765 BRCA patients. For all analyses, patients were grouped into high and low expression cohorts using the Affymetrix probe ID 204812_at. Red lines indicate patients with elevated HCCS expression, while black lines represent those with lower expression. Data were obtained using the Kaplan–Meier Plotter platform (http://www.kmplot.com). (d–g) Subgroup analyses exploring the relationship between HCCS expression and patient survival based on clinical and demographic variables using TCGA datasets via the UALCAN portal (http://ualcan.path.uab.edu/). (d) Survival outcome in relation to race and HCCS expression. (e) Stratification by gender and HCCS expression. (f) Influence of HCCS levels across various breast cancer molecular subtypes. (g) Survival comparison based on menopause status in patients with differing HCCS expression. Statistical significance (*p* value) was assessed using log‐rank tests, with the following annotations: ns = not significant, ∗*p* < 0.05, ∗∗*p* < 0.01, and ∗∗∗*p* < 0.001. The *p* value indicates that there is a statistically significant difference in survival among the groups shown. “Exp.” refers to expression levels, and “med.” denotes medium expression.

**Figure:**
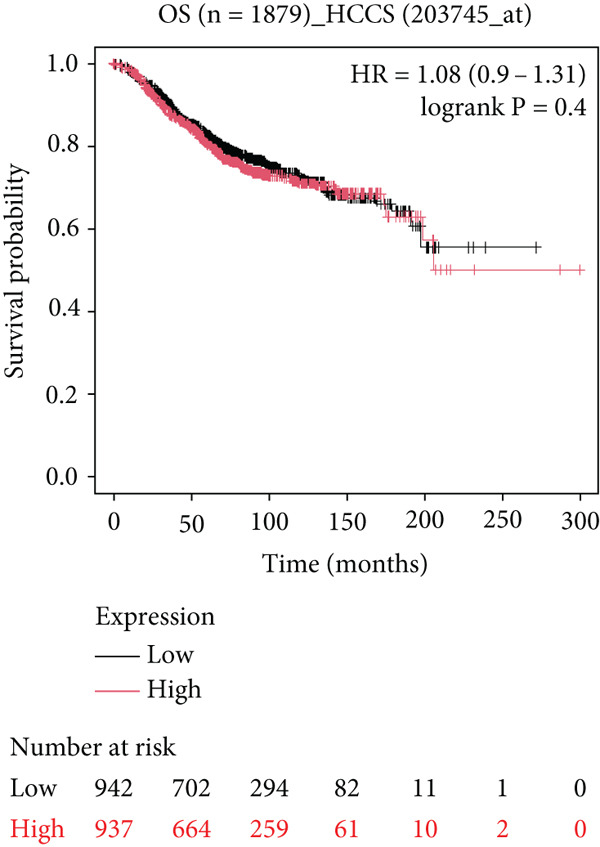
(a)

**Figure:**
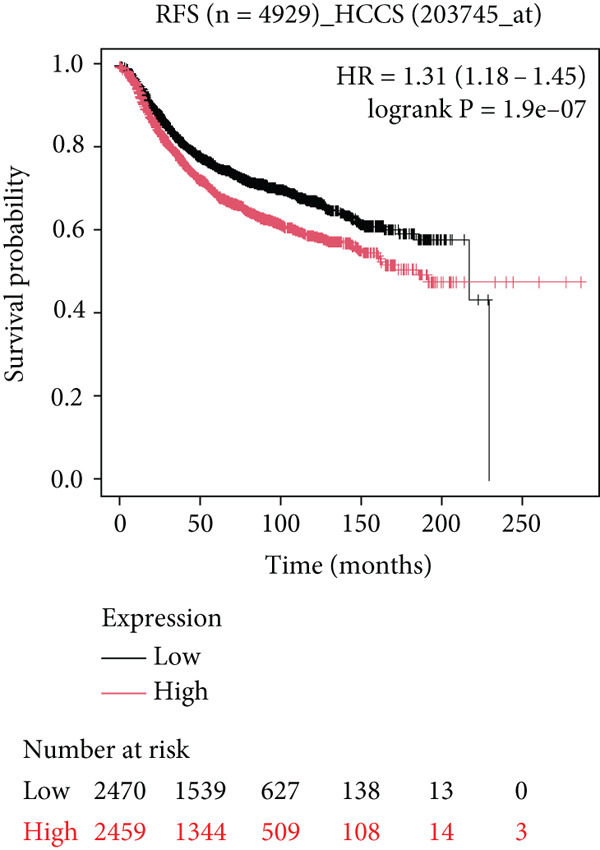
(b)

**Figure:**
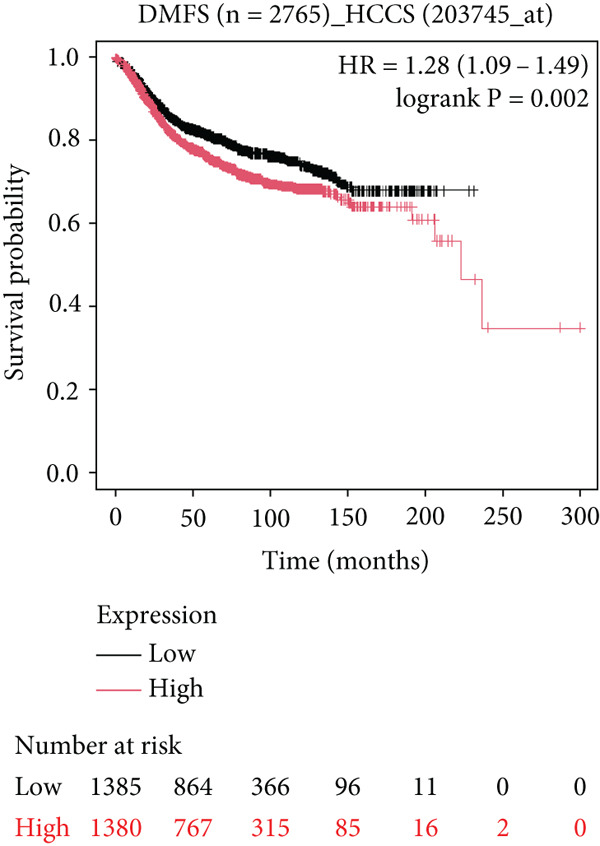
(c)

**Figure:**
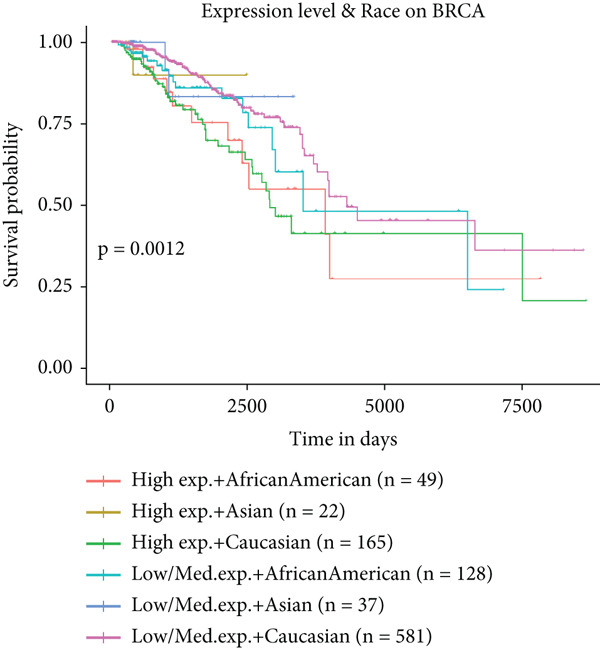
(d)

**Figure:**
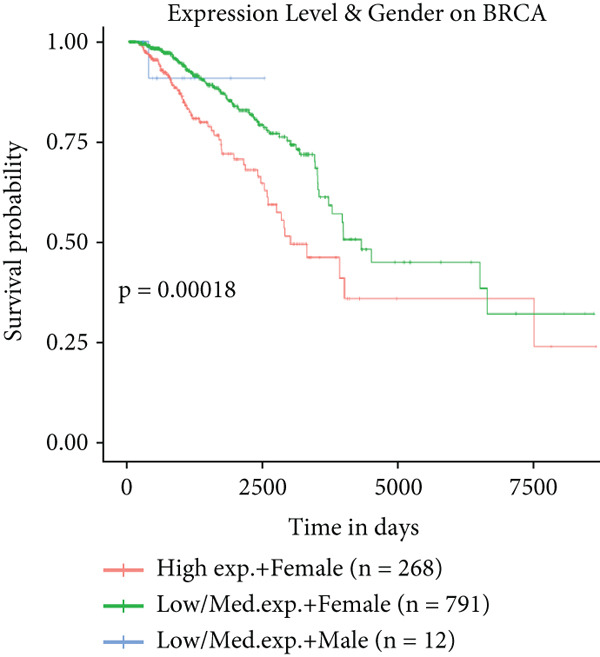
(e)

**Figure:**
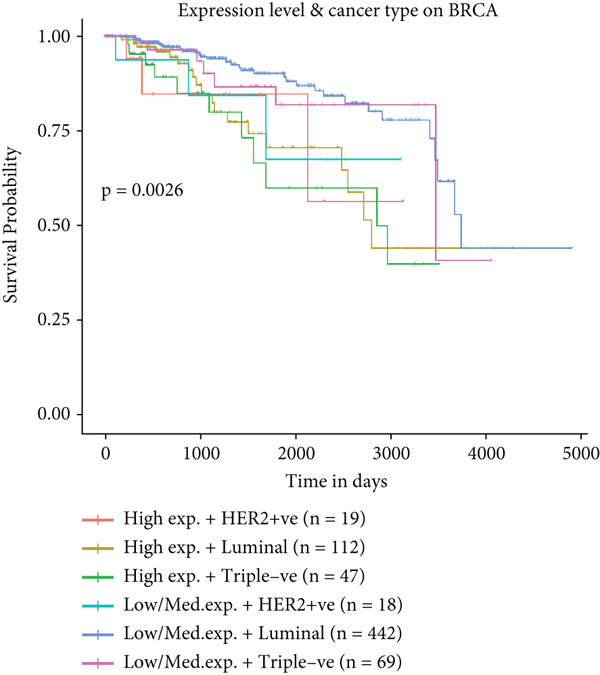
(f)

**Figure:**
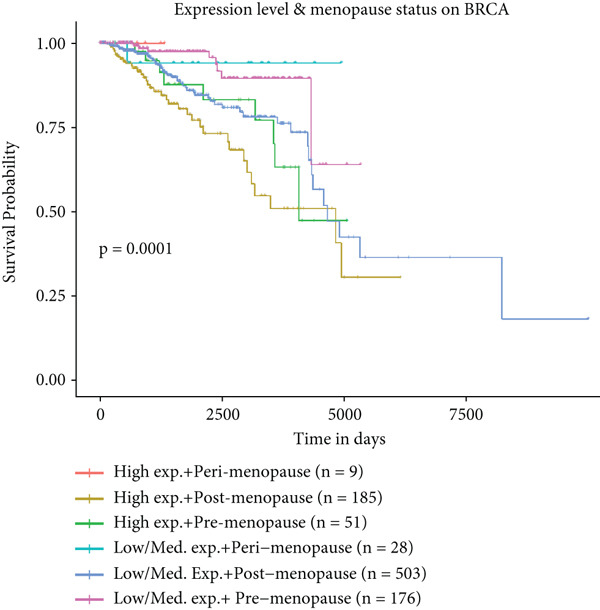
(g)

Conversely, significant associations were observed in the RFS analysis. Patients with lower HCCS expression displayed markedly shorter RFS compared to those with higher expression (*n* = 4929, HR = 1.31 [1.18–1.45], *p* = 1.9e − 07) (Figure 6b). These results imply that high HCCS expression may confer a protective effect in terms of disease recurrence, contrasting with its ambiguous role in OS. Furthermore, DMFS analysis revealed that patients with low HCCS expression demonstrated significantly higher DMFS compared to those with elevated expression (*n* = 2765, HR = 1.28 [1.09–1.49], *p* = 0.002) (Figure 6c), further supporting the prognostic relevance of HCCS in breast cancer progression.

To further investigate the clinical context of HCCS expression, survival analyses were stratified by patient subgroups, including race, gender, cancer subtype, and menopausal status. Among African‐American patients, elevated HCCS expression was significantly associated with reduced survival probability compared to other racial groups (*p* = 0.0012) (Figure 6d). Similarly, in female patients, higher HCCS expression correlated with poorer survival outcomes relative to male patients (*p* = 0.00018) (Figure 6e).

Subtype‐specific analysis revealed that in TNBC, a particularly aggressive subtype, high HCCS expression was significantly linked to reduced survival probability (*p* = 0.0026) (Figure 6f). This finding underscores the potential of HCCS as a prognostic marker in aggressive breast cancer subtypes and highlights its relevance in TNBC.

Additionally, the impact of HCCS expression on survival outcomes in relation to menopausal status was examined. Notably, postmenopausal patients with high HCCS expression exhibited significantly worse survival outcomes compared to premenopausal patients (*p* < 0.0001) (Figure 6g), suggesting a possible role of HCCS in modulating disease progression in postmenopausal breast cancer patients.

Collectively, these findings suggest that HCCS may serve as a significant prognostic biomarker in breast cancer, with distinct implications for relapse‐free and distant metastasis‐free survival. The observed discrepancy between OS and RFS underscores the complexity of HCCS’s prognostic role, warranting further investigation to clarify its utility as a predictive marker in various clinical contexts, particularly in aggressive subtypes like TNBC and in postmenopausal patients.

### 4.6. Correlation of HCCS Expression With Immune Infiltration Profile in Breast Cancer

To investigate the potential immunological implications of HCCS expression in breast cancer, we assessed its correlation with tumor‐infiltrating immune cell populations using Spearman’s correlation analysis on transcriptomic profiles derived from TCGA breast cancer datasets. The immune infiltration data were extracted from established immune deconvolution algorithms, including TIMER, which estimate immune cell abundance from gene expression signatures.

Our results demonstrated modest but statistically significant positive correlations between HCCS expression and the infiltration of multiple immune cell types, including CD8^+^ T cells, CD4^+^ T cells, regulatory T cells (Tregs), memory B cells, neutrophils, M1 macrophages, M2 macrophages, and mast cells (Figures 7a, 7b, 7c, 7d, 7e, 7f, 7g, and 7h). While these associations do not establish causality, our results suggest that HCCS expression may coincide with alterations in both immune‐activating and immunosuppressive components of the tumor microenvironment.

Figure 7Correlation between HCCS expression and immune cell infiltration in breast cancer. Scatter plots illustrate the Spearman correlation between HCCS expression and the abundance of various immune cell populations in breast cancer based on TCGA transcriptomic data. (a) Positive correlation between HCCS expression and CD8^+^ T‐cell infiltration. (b) Positive correlation between HCCS expression and CD4^+^ memory T‐cell infiltration. (c) Positive correlation between HCCS expression and regulatory T‐cell (Treg) infiltration. (d) Positive correlation between HCCS expression and B‐cell infiltration. (e) Positive correlation between HCCS expression and neutrophil infiltration. (f) Positive correlation between HCCS expression and M1 macrophage infiltration. (g) Positive correlation between HCCS expression and M2 macrophage infiltration. (h) Positive correlation between HCCS expression and mast cell infiltration. Correlation strength and significance were determined using Spearman’s rank correlation test, with *p* values < 0.05 considered statistically significant.

**Figure:**
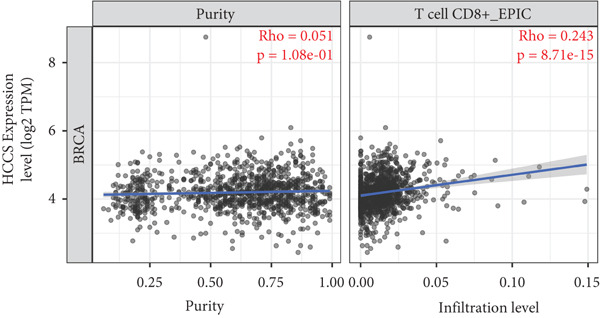
(a)

**Figure:**
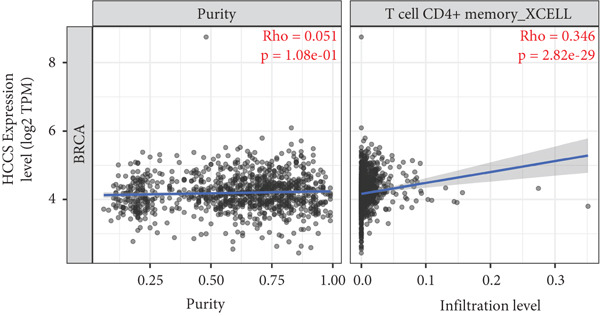
(b)

**Figure:**
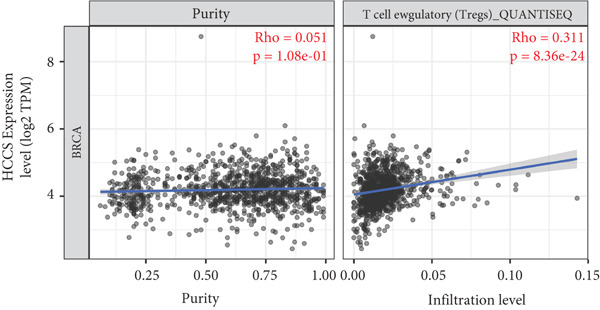
(c)

**Figure:**
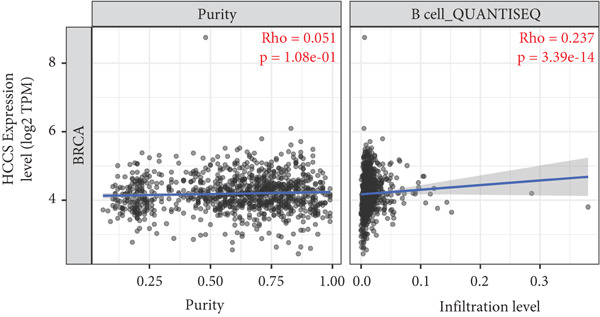
(d)

**Figure:**
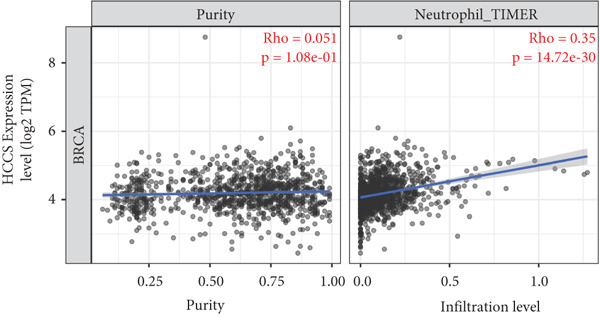
(e)

**Figure:**
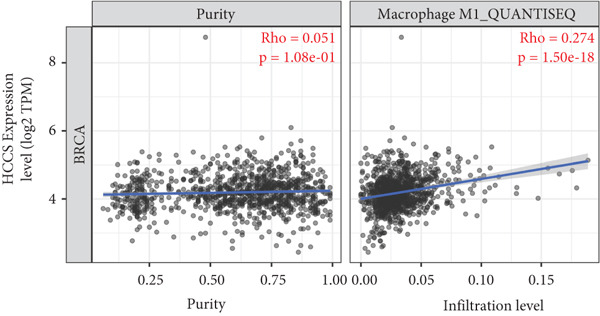
(f)

**Figure:**
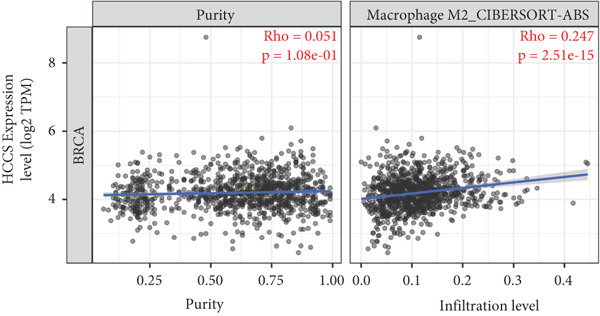
(g)

**Figure:**
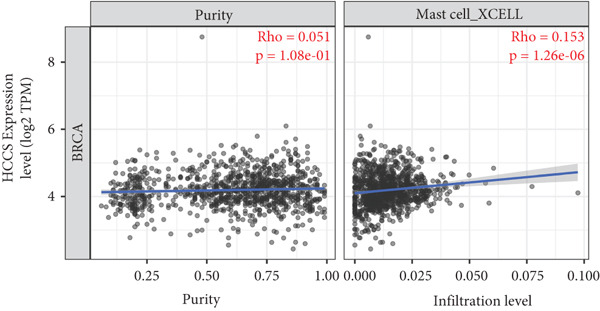
(h)

To assess whether these trends varied across molecular subtypes of breast cancer, we conducted subtype‐specific analyses, including basal‐like, HER2‐enriched, luminal A, and luminal B tumors. Consistent but variable correlation patterns were observed across subtypes (Figure S3), indicating that the association between HCCS and immune infiltration may be subtype‐dependent and influenced by broader tumor‐intrinsic features.

Given the multifactorial regulation of immune cell infiltration in tumors—including cytokine signaling, stromal remodeling, and metabolic reprogramming—the observed correlations should be interpreted cautiously. While the data imply a potential link between HCCS expression and immune modulation, further functional studies are required to delineate whether HCCS plays an active role in recruiting or shaping immune cell populations, or if its expression is reflective of broader oncogenic or metabolic shifts within the tumor.

In summary, these findings highlight a possible association between HCCS expression and the immune landscape of breast cancer, but additional experimental validation is necessary to confirm mechanistic involvement. Future work exploring the immunomodulatory potential of HCCS—particularly in aggressive subtypes like TNBC—could provide valuable insight into its functional relevance in the tumor microenvironment.

### 4.7. Construction and Analysis of Gene and Protein Interaction Networks for HCCS

To further explore the molecular role of HCCS in cancer, we examined its gene and protein interaction networks, aiming to elucidate its broader biological functions and the regulatory pathways it influences. Genes, particularly those like HCCS, do not operate in isolation but interact within complex networks that regulate critical cellular processes such as cell cycle progression, mitotic regulation, and tumorigenesis. Understanding these interaction networks is crucial for comprehending how HCCS contributes to cancer development and progression.

We began by conducting gene correlation analyses to identify genes co‐expressed with HCCS in breast cancer tissues. Our results revealed a set of genes positively correlated with HCCS expression, suggesting that HCCS may play a role in cooperative signaling pathways that promote tumor growth, mitotic stability, and metastasis (Figure 8a). Additionally, we identified genes negatively correlated with HCCS expression, which could indicate opposing regulatory mechanisms or involvement in distinct cellular processes that counteract HCCS’s oncogenic functions (Figure 8b). These findings provide valuable insights into the complex regulatory network in which HCCS is embedded and highlight its potential involvement in key pathways associated with cancer progression.

Figure 8Gene expression correlation analysis of HCCS in BRCA. (a) Expression profile of HCCS in breast adenocarcinoma, accompanied by a heat map displaying the top genes positively correlated with HCCS expression in BRCA samples. (b) Heat map illustrating the top genes negatively correlated with HCCS expression in BRCA. Correlation analysis was based on TCGA BRCA transcriptomic data, with statistically significant (*p* < 0.05) relationships identified using Pearson correlation coefficients.

**Figure:**
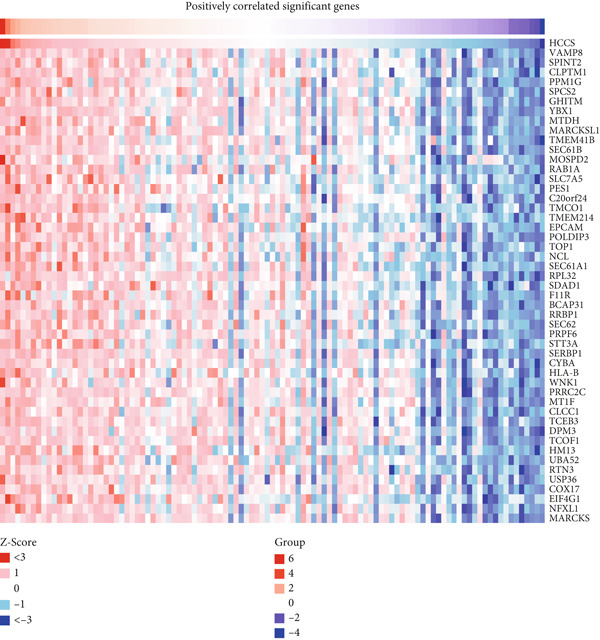
(a) Positively correlated significant genes

**Figure:**
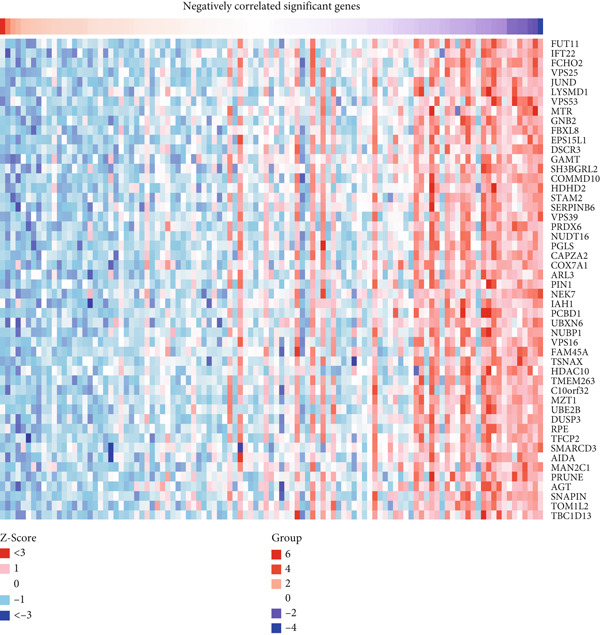
(b) Negatively correlated significant genes

Given the upregulation of HCCS in multiple cancers, particularly in breast cancer and TNBC, constructing these gene interaction networks enhances our understanding of its role in oncogenesis. Integrating these findings with known cancer‐related pathways may also reveal potential therapeutic targets for intervention. Further research, including functional assays and pathway enrichment analyses, is needed to validate these interactions and explore their mechanistic significance in tumor development.

Additionally, to investigate HCCS’s protein‐level interactions, we constructed a PPI network using data from the STRING database. Protein interactions are essential for cellular regulation, and identifying key interactors of HCCS can provide further insights into the biological pathways it influences. Our PPI network analysis revealed a series of high‐confidence protein interactions, including key proteins such as COX10 (score = 0.961), FECH (score = 0.930), ARHGAP6 (score = 0.929), HMOX2 (score = 0.915), HMOX1 (score = 0.906), COX15 (score = 0.889), CYCS (score = 0.825), CYC1 (score = 0.807), GRPR (score = 0.646), and TIMMDC1 (score = 0.639) (Figure S4). These interactors are likely involved in critical cellular processes and may offer insight into the functional role of HCCS in cancer.

Together, these analyses of gene and protein interactions underscore the importance of HCCS in cancer biology, especially in aggressive cancer subtypes like TNBC. The identification of these interactions lays the groundwork for future studies focused on developing HCCS‐targeted therapeutic strategies.

### 4.8. Gene Set Enrichment Analysis of HCCS‐correlated Genes

Enrichment analysis of the 11 HCCS‐correlated genes, identified from a PPI network analysis (Figure S4), revealed a highly significant and coherent set of biological functions, processes, and pathways, primarily centered on mitochondrial heme metabolism and oxidative phosphorylation (Table S1). The most significantly enriched MF were heme binding (*p*_adj_ = 1.82E − 11) and tetrapyrrole binding (*p*_adj_ = 1.82E − 11), with genes including HCCS, FECH, HMOX1, HMOX2, and COX15. Key enzymatic activities such as ferrochelatase activity (FECH), holocytochrome‐c synthase activity (HCCS), and heme oxygenase activity (HMOX1/2) were also significantly enriched. Accordingly, BP analysis highlighted essential roles in heme metabolic process (*p*_adj_ = 5.85E − 09), tetrapyrrole metabolic process (*p*_adj_ = 9.37E − 09), and heme A biosynthetic process (*p*_adj_ = 9.95E − 07). Crucially, these processes are directly linked to mitochondrial respiration, as evidenced by the enrichment of terms like cytochrome complex assembly (*p*_adj_ = 3.75E − 05), cellular respiration (*p*_adj_ = 1.14E − 04), and oxidative phosphorylation (*p*_adj_ = 2.14E − 02). CC analysis strongly localized the gene products to the mitochondrial inner membrane (*p*_adj_ = 2.51E − 08) and mitochondrial envelope (*p*_adj_ = 2.19E − 08), consistent with their roles in electron transport and energy production.

KEGG pathway analysis corroborated these findings, identifying porphyrin metabolism (*p*_adj_ = 4.80E − 11) as the top pathway, followed by oxidative phosphorylation (*p*_adj_ = 1.41E − 04) and metabolic pathways (*p*_adj_ = 1.41E − 04). Notably, the GRPR gene was enriched in processes related to behavior and angiogenesis, while ARHGAP6 was linked to phospholipase regulation, suggesting ancillary roles in signaling.

### 4.9. Validation of HCCS Expression in Independent Datasets

To confirm the expression profile of HCCS, we analyzed the GSE65194 dataset consisting of 167 breast tumors and 11 normal breast tissues. Consistent with our discovery cohort, HCCS was significantly upregulated in breast tumors compared with normal tissues (*p* = 5.192E − 07, Student’s *t* test) (Figure 9a). Further exploration of HCCS expression using the GOBO platform demonstrated subtype‐specific variation. HCCS expression was significantly higher in the basal, HER2‐enriched, and luminal B subtype, while downregulated in lum A and normal‐like subtype (*p* < 0.00001) (Figure 9b). The patients with an ER‐negative status exhibited a higher HCCS expression than those with an ER‐positive status (*p* < 0.00001) (Figure 9c). The highest HCCS transcript expression was observed in grade 3 tumors, compared with grade 1 and grade 2 tumors (*p* < 0.00001) (Figure 9d).

Figure 9Validation of HCCS expression in an independent dataset, across breast cancer molecular subtypes and clinicopathological features. (a) Boxplot showing HCCS expression levels in breast tumor tissues versus normal breast tissues based on the GSE65194 dataset. (b) Expression of HCCS in different breast cancer molecular subtypes. (c) Comparison of HCCS expression between estrogen receptor (ER)‐positive and ER‐negative tumors. (d) Expression of HCCS across histological grades.

**Figure:**
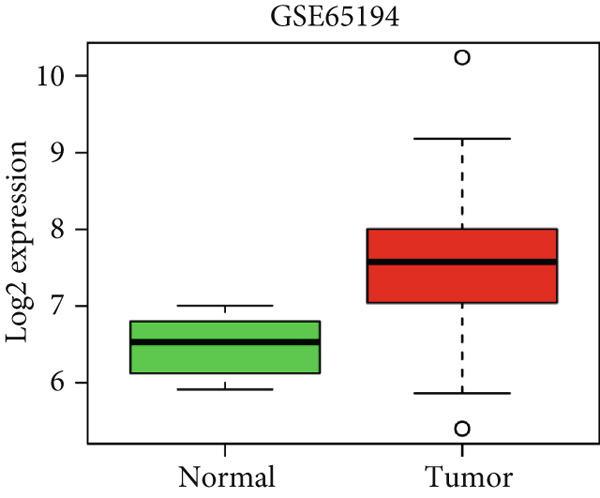
(a)

**Figure:**
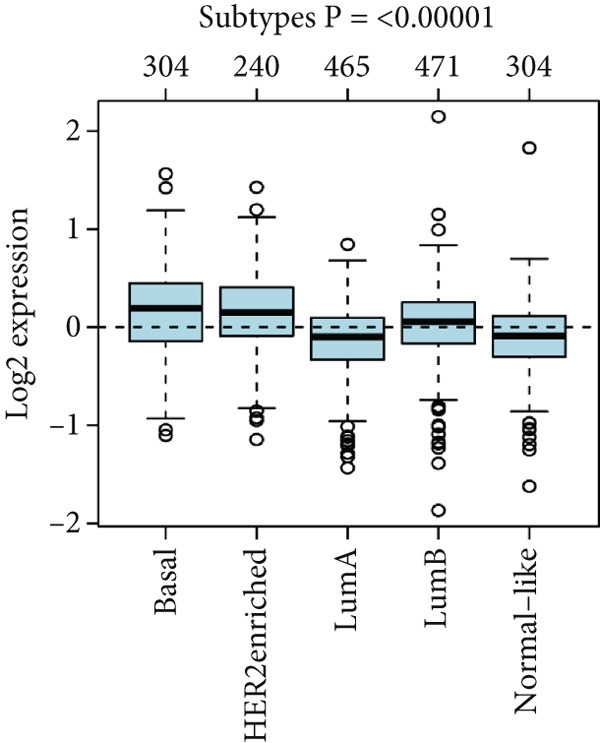
(b)

**Figure:**
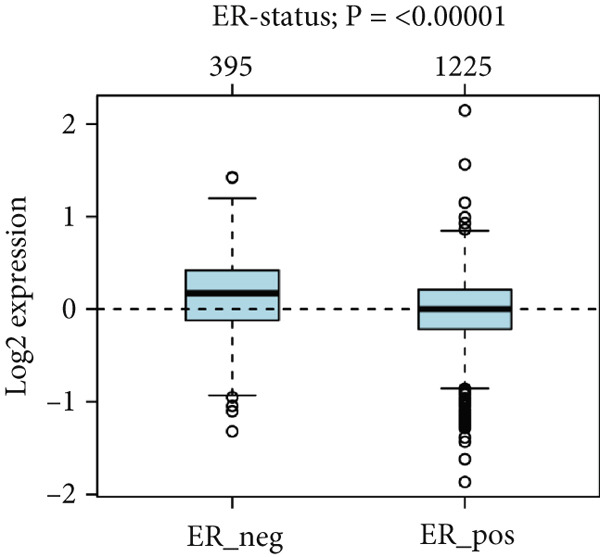
(c)

**Figure:**
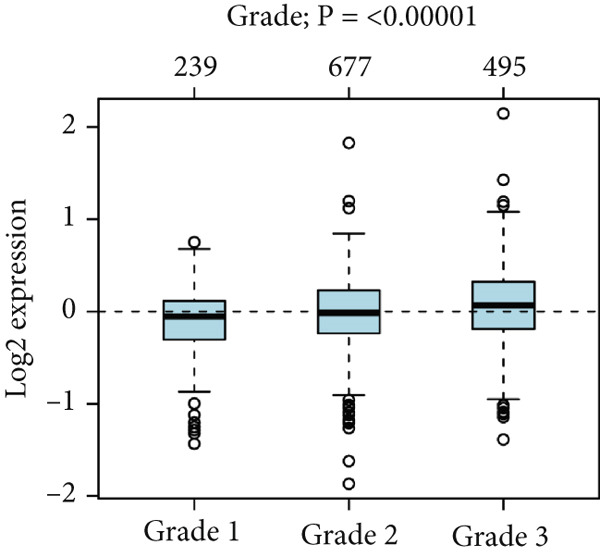
(d)

### 4.10. Validation of Prognostic Significance of HCCS in Breast Cancer

Using the GOBO outcome module, we further assessed the prognostic relevance of HCCS expression across breast cancer subgroups. Patients with elevated HCCS expression exhibited significantly poorer OS across all breast cancer subtypes (*p* = 0.038) (Figure 10a). Similarly, high and medium HCCS expression levels were associated with worse DMFS (*p* = 4E − 04) in most subtypes (Figure 10c), while increased HCCS expression also correlated with RFS (*p* = 0.00019) compared with patients with lower expression levels (Figure 10e). Multivariate Cox regression analysis, adjusted for key clinicopathological variables including ER status, nodal status, grade, age, and tumor size, further confirmed that HCCS expression was an independent predictor of poor outcomes in breast cancer (Figures 10b, 10d, and 10f). Collectively, these findings highlight that higher HCCS mRNA expression is consistently associated with poor prognosis in breast cancer, in agreement with our earlier analyses.

Figure 10Prognostic significance of HCCS in breast cancer (GOBO outcome module). (a,c, e) Kaplan–Meier survival curves illustrating the association of HCCS expression with OS, DMFS, and RFS, respectively. Patients were stratified into three quantiles (low, medium, high HCCS expression). *p* Values were determined using the log‐rank test. (b,d,f) Cox multivariate proportional hazards models adjusting for ER status, nodal status, grade, age, and tumor size as covariates and using OS, DMFS, RFS as the endpoint with 10‐year censoring.

**Figure:**
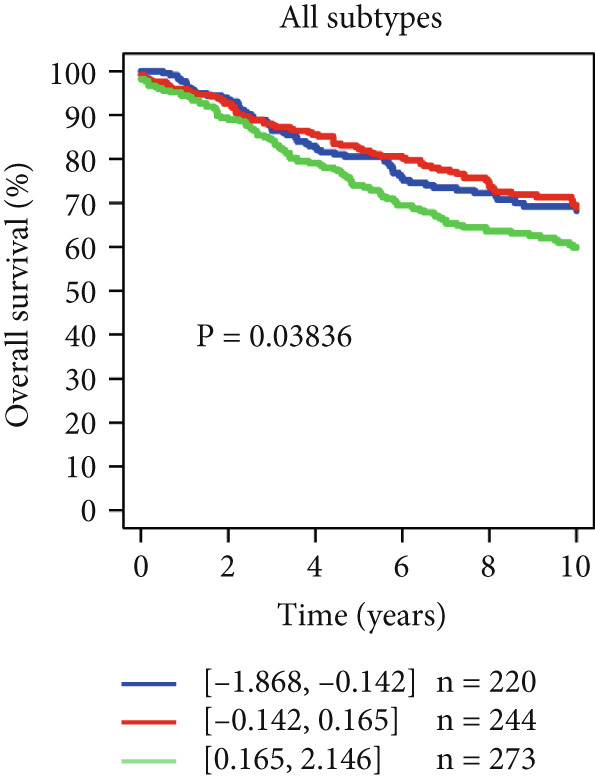
(a)

**Figure:**
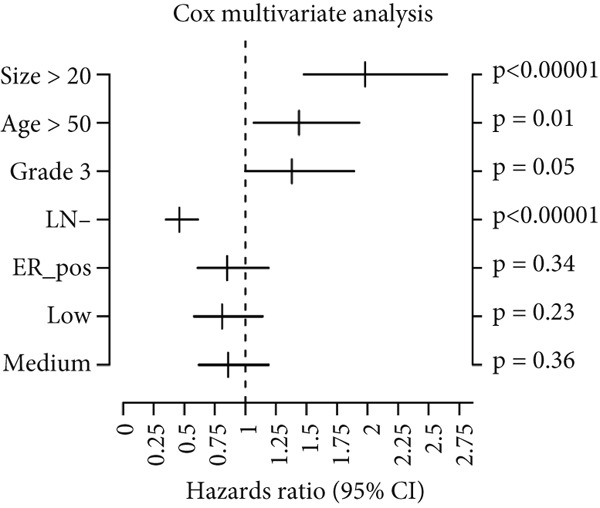
(b)

**Figure:**
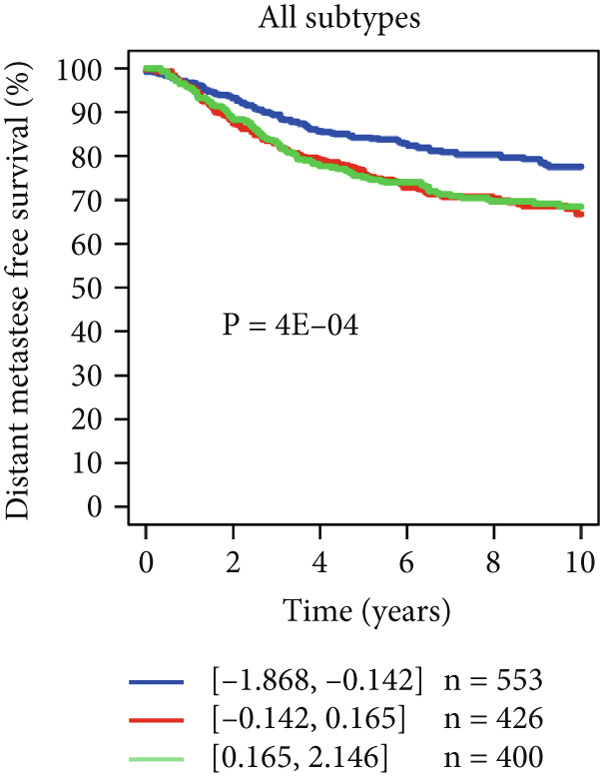
(c)

**Figure:**
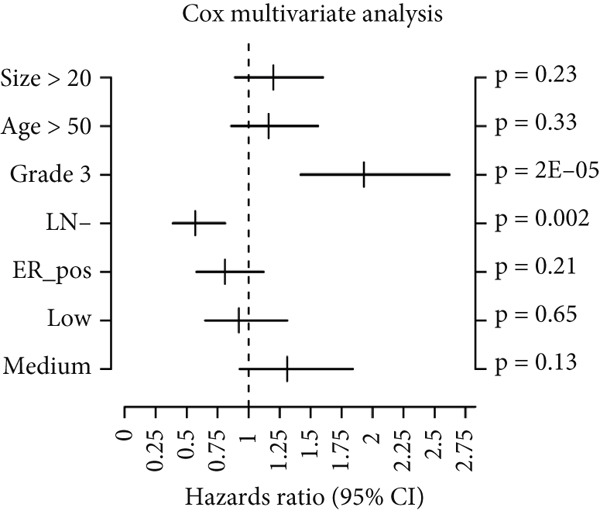
(d)

**Figure:**
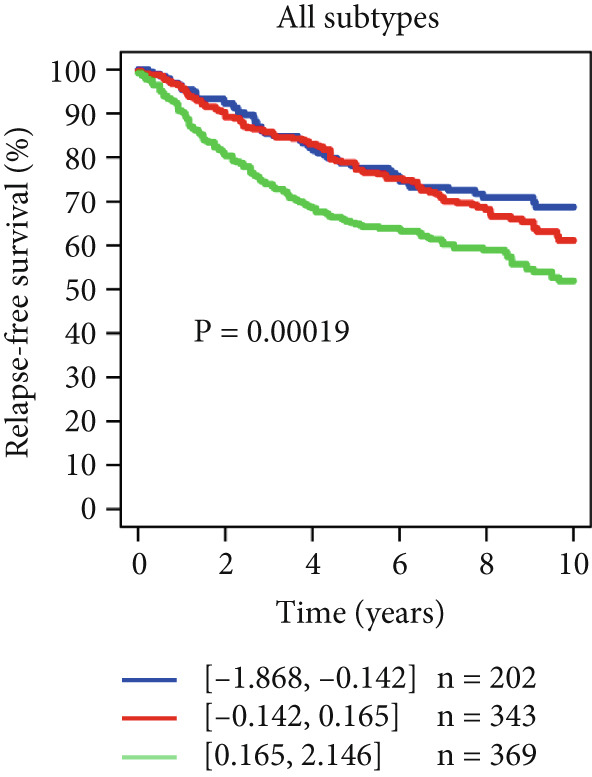
(e)

**Figure:**
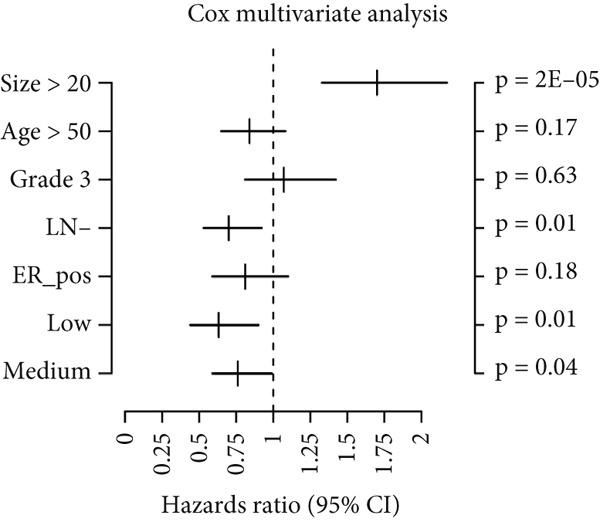
(f)

## 5. Discussion

In this study, we demonstrated the significant role of HCCS in the pathogenesis of breast cancer, particularly in aggressive subtypes such as TNBC. HCCS was found to be markedly upregulated in breast tumors, where its expression correlated with key clinicopathological features including nodal metastasis, stage, and patient prognosis. These findings suggest that HCCS may serve not only as a biomarker of disease progression but also as a potential therapeutic target, especially in TNBC, which remains challenging due to its poor prognosis and limited treatment options [5, 42].

HCCS, a mitochondrial enzyme essential for cytochrome c maturation, has traditionally been studied in the context of oxidative phosphorylation and apoptosis [15]. Merging evidence, however, indicates that HCCS also influences inflammatory responses, redox balance, and immune regulation, expanding its role in cancer biology beyond energy metabolism [10, 11]. Our analysis revealed a robust association between elevated HCCS expression and reduced RFS, with the strongest effects observed in aggressive breast cancer subtypes. Mechanistically, this may reflect HCCS‐mediated dysregulation of cytochrome c processing, which disrupts apoptotic priming and enhances tumor cell persistence. In addition, aberrant HCCS activity could alter mitochondrial ROS generation, fostering a pro‐inflammatory microenvironment that supports tumor recurrence. Interestingly, while HCCS expression was a strong predictor of recurrence risk, its relationship with OS was less consistent, suggesting that HCCS is more relevant to early relapse than late‐stage mortality. We further evaluated the prognostic relevance of HCCS expression across breast cancer and its subtypes using independent datasets and found that elevated HCCS mRNA levels were consistently associated with poor prognosis. These findings position HCCS as a potentially important biomarker for recurrence risk assessment, particularly in aggressive breast cancer subtypes.

We further examined the relationship between HCCS and the tumor immune microenvironment. High HCCS expression correlated with infiltration of multiple immune cell types, including CD8+ T cells, regulatory T cells (Tregs), and macrophages. Although CD8+ T cells are typically cytotoxic, their positive correlation with HCCS expression may reflect impaired functionality in the context of an immunosuppressive tumor microenvironment. The simultaneous enrichment of Tregs further supports the idea that HCCS overexpression contributes to immune evasion and tumor progression. Moreover, the stronger association with M2 macrophages compared to M1 macrophages suggests that HCCS may favor an immunosuppressive, pro‐tumorigenic milieu, particularly in TNBC. These observations align with reports that mitochondrial regulators can shape immune cell polarization and antitumor immunity [16, 43].

Beyond immune modulation, our study suggests that HCCS may interact with cellular stress regulators such as heme oxygenase‐1 (HO‐1), a critical enzyme in redox balance. Through its role in mitochondrial heme metabolism, HCCS may influence HO‐1 activity and thereby impact ROS production, oxidative stress, and tumor progression. Dysregulation of this HCCS–HO‐1 axis could promote tumor survival by enhancing oxidative adaptation while simultaneously driving genomic instability. This duality may explain why elevated HCCS is consistently linked with poor prognosis in breast cancer.

Epigenetic dysregulation also emerged as a potential mechanism underlying HCCS overexpression. Our methylation analysis revealed promoter hypomethylation of HCCS in breast cancer tissues, a common feature of oncogene activation [14, 44]. This finding is consistent with prior studies highlighting the importance of epigenetic alterations in regulating mitochondrial gene expression [45]. Additionally, we identified a network of genes co‐expressed with HCCS, both positively and negatively, suggesting that HCCS might engage in complex molecular pathways associated with tumor growth and metastasis. The PPI network analysis further revealed key interactors such as COX10 and FECH, which are involved in mitochondrial function and heme metabolism, supporting the idea that HCCS could influence mitochondrial dynamics and cellular energy production in cancer cells [11, 46].

Our GSEA results establish HCCS and its network as central to a critical metabolic axis in breast cancer, governing heme biosynthesis, cytochrome c maturation, and mitochondrial bioenergetics. The significant enrichment of heme metabolism genes—including HCCS, FECH, and ∗COX10/15∗—indicates an adaptive mechanism to upregulate mitochondrial energy production and fuel proliferation [47]. This reliance on oxidative phosphorylation is a recognized cancer hallmark [48, 49]. The co‐enrichment of heme synthesis (FECH, COX10, and COX15) and degradation (HMOX1, HMOX2) genes reveals a tightly regulated homeostasis circuit. HMOX1 degradation yields both antioxidant products that promote survival and free iron that can potentiate ferroptosis [46, 50]. This dual role positions heme metabolism as a therapeutic target to disrupt cancer cell balance [51]. The strong mitochondrial localization underscores this network’s role as a potential vulnerability; disrupting heme supply could induce metabolic catastrophe in dependent cells. Finally, the correlation of GRPR (a known oncogene driving proliferation and angiogenesis) [52] with this metabolic core suggests a compelling link between mitogenic signaling and mitochondrial bioenergetics in breast cancer.

## 6. Limitations

Despite the comprehensive analyses conducted, several limitations must be acknowledged in this study. First, the reliance on publicly available datasets such as TCGA and the Kaplan–Meier Plotter may introduce selection bias and limit the generalizability of our findings to broader patient populations. Additionally, the study predominantly utilized mRNA expression data without validating the observed associations at the protein level, which may overlook post‐transcriptional regulatory mechanisms. A major limitation of our study is the lack of functional assays directly validating the role of HCCS in tumorigenesis. In particular, we did not perform siRNA‐mediated knockdown of HCCS in breast cancer cell lines to evaluate the resulting phenotypic effects. Future experimental work involving HCCS silencing and overexpression assays needs to validate its involvement in cancer cell proliferation, apoptosis, chemoresistance, and mitochondrial function. Such experiments would offer more definitive evidence linking HCCS to breast cancer progression and its potential as a therapeutic target for further investigation. The mechanistic pathways underlying the observed prognostic effects of HCCS expression in aggressive subtypes such as TNBC was not explored, warranting further in vitro and in vivo studies to elucidate the biological role of HCCS in breast cancer progression. Finally, the study did not explore the underlying molecular mechanisms linking HCCS expression to breast cancer progression, highlighting the need for further functional studies to elucidate the biological pathways involved. Future studies involving wet‐lab experiments and analysis of clinical samples will be crucial to validate and expand our findings, thereby enhancing their translational relevance for BRCA diagnostics and therapeutic applications.

## 7. Conclusion

Our study provides compelling evidence that HCCS is a critical player in breast cancer progression and may serve as a novel biomarker and therapeutic target. HCCS is significantly overexpressed in various cancers, particularly in breast cancer and triple‐negative breast cancer (TNBC). Promoter hypomethylation may contribute to HCCS overexpression, while mutation analysis reveals potential oncogenic alterations. HCCS is associated with immune cell infiltration, indicating its role in modulating the tumor microenvironment. Additionally, gene and protein interaction networks highlight HCCS’s involvement in mitochondrial function and apoptotic regulation. The gene set enrichment analysis reveals that HCCS and its correlated genes are not random associates but form a coherent functional module integral to breast cancer cell biology. The primary pathway identified is the mitochondrial heme metabolism and oxidative phosphorylation pathway, which is crucial for meeting the high‐energy demands of proliferating cancer cells. These findings suggest that HCCS may serve as a promising prognostic biomarker and therapeutic target in aggressive breast cancer subtypes. However, further studies should experimentally validate these findings and elucidate the precise molecular mechanisms by which HCCS contributes to breast cancer pathogenesis.

**Nomenclature**
TCGA: The Cancer Genome Atlas
GTEx: Genotype‐tissue expression
HCCS: Holocytochrome c synthase
ACC: Adrenocortical carcinoma
BLCA: Bladder urothelial carcinoma
BRCA: Breast invasive carcinoma
CESC: Cervical squamous cell carcinoma and endocervical adenocarcinoma
CHOL: Cholangio carcinoma
COAD: Colon adenocarcinoma
DLBC: Lymphoid neoplasm diffuse large B‐cell lymphoma
ESCA: Esophageal carcinoma
GBM: Glioblastoma multiforme
GEO: Gene Expression Omnibus
GOBO: Gene expression‐based Outcome for Breast cancer Online
HNSC: Head and neck squamous cell carcinoma
KICH: Kidney chromophobe
KIRC: Kidney renal clear cell carcinoma
KIRP: Kidney renal papillary cell carcinoma
LAML: Acute myeloid leukemia
LGG: Brain lower grade glioma
LIHC: Liver hepatocellular carcinoma
LUAD: Lung adenocarcinoma
LUSC: Lung squamous cell carcinoma
MESO: Mesothelioma
OV: Ovarian serous cystadenocarcinoma
PAAD: Pancreatic adenocarcinoma
PCPG: Pheochromocytoma and paraganglioma
PRAD: Prostate adenocarcinoma
READ: Rectum adenocarcinoma
SARC: Sarcoma
SKCM: Skin cutaneous melanoma
STAD: Stomach adenocarcinoma
TGCT: Testicular germ cell tumors
THCA: Thyroid carcinoma
THYM: Thymoma
UCEC: Uterine corpus endometrial carcinoma
UCS: Uterine carcinosarcoma
UVM: Uveal melanoma

## Ethics Statement

This study is a computational biology research study and is exempt from institutional review board approval.

## Consent

The authors have nothing to report.

## Conflicts of Interest

The authors declare no conflicts of interest.

## Author Contributions

Sm Faysal Bellah: conceptualization, methodology, formal analysis, validation, writing — original draft, supervision. Md Alim Hossen: methodology, formal analysis, validation, writing — review and editing, S M Saker Billah: methodology, formal analysis, writing — review and editing. Md Nur Islam: methodology, formal analysis, writing — review and editing.

## Funding

No funding was received for this manuscript.

## Supporting information


**Supporting Information** Additional supporting information can be found online in the Supporting Information section. Figure S1. Overview of HCCS gene expression in different human tissue samples. Figure S2. Differential HCCS expression in TCGA cancer data sets. Figure S3. HCCS expression with immune infiltration profile in breast cancer subtypes. Figure S4. Protein–protein interaction network of HCCS.

## Data Availability

On reasonable request, the corresponding author will provide the datasets used in and/or analyzed during the current study.

## References

[1] Sung H. , Ferlay J. , Siegel R. L. , Laversanne M. , Soerjomataram I. , Jemal A. , and Bray F. , Global Cancer Statistics 2020: GLOBOCAN Estimates of Incidence and Mortality Worldwide for 36 Cancers in 185 Countries, CA: A Cancer Journal for Clinicians. (2021) 71, no. 3, 209–249, 10.3322/caac.21660, 33538338. 33538338

[2] Bray F. , Laversanne M. , Sung H. , Ferlay J. , Siegel R. L. , Soerjomataram I. , and Jemal A. , Global Cancer Statistics 2022: GLOBOCAN Estimates of Incidence and Mortality Worldwide for 36 Cancers in 185 Countries, CA: A Cancer Journal for Clinicians. (2024) 74, no. 3, 229–263, 10.3322/caac.21834, 38572751. 38572751

[3] Bellah S. F. , Salam M. A. , Karim M. R. , Hossain M. J. , and Ashrafudoulla M. , Epidemiology of Breast Cancer Among the Female Patients in Bangladesh, Oriental Pharmacy and Experimental Medicine. (2016) 16, no. 2, 85–95, 10.1007/s13596-016-0225-y, 2-s2.0-84975678633.

[4] Hartwell L. H. and Kastan M. B. , Cell Cycle Control and Cancer, Science. (1994) 266, no. 5192, 1821–1828, 10.1126/science.7997877, 2-s2.0-0028568315. 7997877

[5] Medina M. A. , Oza G. , Sharma A. , Arriaga L. G. , Hernández Hernández J. M. , Rotello V. M. , and Ramirez J. T. , Triple-Negative Breast Cancer: A Review of Conventional and Advanced Therapeutic Strategies, International Journal of Environmental Research and Public Health. (2020) 17, no. 6, 10.3390/ijerph17062078. PMC7143295 32245065

[6] Bertucci F. , Finetti P. , and Birnbaum D. , Basal Breast Cancer: A Complex and Deadly Molecular Subtype, Current Molecular Medicine. (2012) 12, no. 1, 96–110, 10.2174/156652412798376134, 2-s2.0-83455236666, 22082486. 22082486 PMC3343384

[7] Shao M.-T. , Hu Y.-Z. , Ding H. , Wu Q. , Pan J.-H. , Zhao X.-X. , and Pan Y.-L. , The Overexpression of ZWINT in Integrated Bioinformatics Analysis Forecasts Poor Prognosis in Breast Cancer, Translational Cancer Research. (2020) 9, no. 1, 187–193, 10.21037/tcr.2019.12.66, 35117172. 35117172 PMC8798864

[8] Bellah S. F. , Sonia F. A. , Ferdous M. R. , Durojaye O. A. , and Islam M. R. , CCL18 and EGF May Serve as Potential Prognostic Biomarkers and Therapeutic Targets for Human Breast Cancer, International Journal of Breast Cancer. (2025) 2025, no. 1, 8856457, 10.1155/ijbc/8856457, 40692603. 40692603 PMC12279427

[9] Bellah S. , Akbar H. , Billah S. , and Sedzro D. , The Role of CCL18 protein in Breast Cancer Development and Progression, Cell Biology: Research & Therapy. (2018) 7, no. 1, 10.4172/2324-9293.1000138.

[10] Chatterjee A. , Dasgupta S. , and Sidransky D. , Mitochondrial Subversion in Cancer, Cancer Prevention Research. (2011) 4, no. 5, 638–654, 10.1158/1940-6207.CAPR-10-0326, 2-s2.0-79955835149, 21543342. 21543342 PMC3298745

[11] Babbitt S. E. , San Francisco B. , Mendez D. L. , Lukat-Rodgers G. S. , Rodgers K. R. , Bretsnyder E. C. , and Kranz R. G. , Mechanisms of Mitochondrial Holocytochrome c Synthase and the Key Roles Played by Cysteines and Histidine of the Heme Attachment Site, Cys-XX-Cys-His, Journal of Biological Chemistry. (2014) 289, no. 42, 28795–28807, 10.1074/jbc.M114.593509, 2-s2.0-84908032075, 25170082. 25170082 PMC4200240

[12] San Francisco B. , Bretsnyder E. C. , and Kranz R. G. , Human Mitochondrial Holocytochrome c Synthase′s Heme Binding, Maturation Determinants, and Complex Formation With Cytochrome c, Proceedings of the National Academy of Sciences of the United States of America. (2013) 110, no. 9, E788–E797, 10.1073/pnas.1213897109, 2-s2.0-84874480835, 23150584. 23150584 PMC3587199

[13] Wang S. F. , Tseng L. M. , and Lee H. C. , Role of Mitochondrial Alterations in Human Cancer Progression and Cancer Immunity, Journal of Biomedical Science. (2023) 30, no. 1, 10.1186/s12929-023-00956-w, 37525297. PMC10392014 37525297

[14] Baylin S. B. and Jones P. A. , A Decade of Exploring the Cancer Epigenome - Biological and Translational Implications, Nature Reviews Cancer. (2011) 11, no. 10, 726–734, 10.1038/nrc3130, 2-s2.0-80053144962, 21941284. 21941284 PMC3307543

[15] Luo P. , Wu S. , Li Z. , Tan S. , Zeng L. , Tang Y. , Luo L. , Li Y. , and Tang Z. , Pathological Significance and Therapeutic Prospects of HCCS Expression Patterns in Lung Adenocarcinoma, Cell Adhesion & Migration. (2025) 19, no. 1, 2520632, 10.1080/19336918.2025.2520632, 40611816. 40611816 PMC12233882

[16] Hinshaw D. C. and Shevde L. A. , The Tumor Microenvironment Innately Modulates Cancer Progression, Cancer Research. (2019) 79, no. 18, 4557–4566, 10.1158/0008-5472.CAN-18-3962, 2-s2.0-85072234203, 31350295. 31350295 PMC6744958

[17] van Rahden V. A. , Rau I. , Fuchs S. , Kosyna F. K. , de Almeida H. L. , Fryssira H. , Isidor B. , Jauch A. , Joubert M. , Lachmeijer A. M. A. , Zweier C. , Moog U. , and Kutsche K. , Clinical Spectrum of Females With HCCS Mutation: From No Clinical Signs to a Neonatal Lethal Form of the Microphthalmia With Linear Skin Defects (MLS) Syndrome, Orphanet Journal of Rare Diseases. (2014) 9, no. 1, 10.1186/1750-1172-9-53, 2-s2.0-84899560247, 24735900. PMC4021606 24735900

[18] Wimplinger I. , Morleo M. , Rosenberger G. , Iaconis D. , Orth U. , Meinecke P. , Lerer I. , Ballabio A. , Gal A. , Franco B. , and Kutsche K. , Mutations of the Mitochondrial Holocytochrome c-Type Synthase in X-Linked Dominant Microphthalmia With Linear Skin Defects Syndrome, American Journal of Human Genetics. (2006) 79, no. 5, 878–889, 10.1086/508474, 2-s2.0-33751098033, 17033964. 17033964 PMC1698567

[19] Reis L. M. , Basel D. , Bitoun P. , Walton D. S. , Glaser T. , and Semina E. V. , Novel Intragenic and Genomic Variants Highlight the Phenotypic Variability in HCCS-Related Disease, Genes. (2024) 15, no. 12, 10.3390/genes15121636, 39766903. PMC11675438 39766903

[20] Schaefer L. , Ballabio A. , and Zoghbi H. Y. , Cloning and Characterization of a Putative Human Holocytochrome c-Type Synthetase Gene (HCCS) Isolated From the Critical Region for Microphthalmia With Linear Skin Defects (MLS), Genomics. (1996) 34, no. 2, 166–172, 10.1006/geno.1996.0261, 2-s2.0-0029882297, 8661044. 8661044

[21] Wimplinger I. , Shaw G. M. , and Kutsche K. , HCCS Loss-of-Function Missense Mutation in a Female With Bilateral Microphthalmia and Sclerocornea: A Novel Gene for Severe Ocular Malformations?, Molecular Vision. (2007) 13, 1475–1482, 17893649. 17893649

[22] Vyas S. , Zaganjor E. , and Haigis M. C. , Mitochondria and Cancer, Cell. (2016) 166, no. 3, 555–566. 27471965 10.1016/j.cell.2016.07.002 PMC5036969

[23] Reza M. S. , Harun-Or-Roshid M. , Islam M. A. , Hossen M. A. , Hossain M. T. , Feng S. , Xi W. , Mollah M. N. H. , and Wei Y. , Bioinformatics Screening of Potential Biomarkers from mRNA Expression Profiles to Discover Drug Targets and Agents for Cervical Cancer, International Journal of Molecular Sciences. (2022) 23, no. 7, 10.3390/ijms23073968, 35409328. PMC8999699 35409328

[24] Hossen M. A. , Reza M. S. , Harun-Or-Roshid M. , Islam M. A. , Siddika M. A. , and Mollah M. N. H. , Identification of Drug Targets and Agents Associated with Hepatocellular Carcinoma through Integrated Bioinformatics Analysis, Current Cancer Drug Targets. (2023) 23, no. 7, 547–563, 10.2174/1568009623666230214100159, 36786134. 36786134

[25] Reza M. S. , Hossen M. A. , Harun-Or-Roshid M. , Siddika M. A. , Kabir M. H. , and Mollah M. N. H. , Metadata Analysis to Explore Hub of the Hub-Genes Highlighting Their Functions, Pathways and Regulators for Cervical Cancer Diagnosis and Therapies, Discover Oncology. (2022) 13, no. 1, 10.1007/s12672-022-00546-6, 35994213. PMC9395557 35994213

[26] Li T. , Fu J. , Zeng Z. , Cohen D. , Li J. , Chen Q. , Li B. , and Liu X. S. , TIMER2.0 for Analysis of Tumor-Infiltrating Immune Cells, Nucleic Acids Research. (2020) 48, no. W1, W509–w514, 10.1093/nar/gkaa407, 32442275. 32442275 PMC7319575

[27] Bartha Á. and Győrffy B. , TNMplot.com: A Web Tool for the Comparison of Gene Expression in Normal, Tumor and Metastatic Tissues, International Journal of Molecular Sciences. (2021) 22, no. 5, 10.3390/ijms22052622, 33807717. PMC7961455 33807717

[28] GTEx Consortium , The GTEx Consortium Atlas of Genetic Regulatory Effects Across Human Tissues, Science. (2020) 369, no. 6509, 1318–1330, 10.1126/science.aaz1776, 32913098. 32913098 PMC7737656

[29] Carithers L. J. , Ardlie K. , Barcus M. , Branton P. A. , Britton A. , Buia S. A. , Compton C. C. , DeLuca D. S. , Peter-Demchok J. , Gelfand E. T. , Guan P. , Korzeniewski G. E. , Lockhart N. C. , Rabiner C. A. , Rao A. K. , Robinson K. L. , Roche N. V. , Sawyer S. J. , Segrè A. V. , Shive C. E. , Smith A. M. , Sobin L. H. , Undale A. H. , Valentino K. M. , Vaught J. , Young T. R. , Moore H. M. , and on behalf of the GTEx Consortium , A Novel Approach to High-Quality Postmortem Tissue Procurement: The GTEx Project, Biopreservation and Biobanking. (2015) 13, no. 5, 311–319, 10.1089/bio.2015.0032, 2-s2.0-84945292900, 26484571. 26484571 PMC4675181

[30] Chandrashekar D. S. , Karthikeyan S. K. , Korla P. K. , Patel H. , Shovon A. R. , Athar M. , Netto G. J. , Qin Z. S. , Kumar S. , Manne U. , Creighton C. J. , and Varambally S. , UALCAN: An Update to the Integrated Cancer Data Analysis Platform, Neoplasia. (2022) 25, 18–27, 10.1016/j.neo.2022.01.001, 35078134. 35078134 PMC8788199

[31] Bellah S. F. , Salam M. A. , Billah S. M. S. , and Karim M. R. , Genetic Association inCYP3A4andCYP3A5genes Elevate the Risk of Prostate Cancer, Annals of Human Biology. (2023) 50, no. 1, 63–75, 10.1080/03014460.2023.2171122. 36688864

[32] Forbes S. A. , Beare D. , Bindal N. , Bamford S. , Ward S. , Cole C. G. , Jia M. , Kok C. , Boutselakis H. , De T. , Sondka Z. , Ponting L. , Stefancsik R. , Harsha B. , Tate J. , Dawson E. , Thompson S. , Jubb H. , and Campbell P. J. , COSMIC: High-Resolution Cancer Genetics Using the Catalogue of Somatic Mutations in Cancer, Current Protocols in Human Genetics. (2016) 91, no. 1, 10.11.1–10.11.37, 10.1002/cphg.21, 2-s2.0-85002612973, 27727438. 27727438

[33] Forbes S. A. , Beare D. , Boutselakis H. , Bamford S. , Bindal N. , Tate J. , Cole C. G. , Ward S. , Dawson E. , Ponting L. , Stefancsik R. , Harsha B. , Kok C. Y. , Jia M. , Jubb H. , Sondka Z. , Thompson S. , De T. , and Campbell P. J. , COSMIC: Somatic Cancer Genetics at High-Resolution, Nucleic Acids Research. (2017) 45, no. D1, D777–d783, 10.1093/nar/gkw1121, 2-s2.0-85016154270, 27899578. 27899578 PMC5210583

[34] Győrffy B. , Surowiak P. , Budczies J. , and Lánczky A. , Online Survival Analysis Software to Assess the Prognostic Value of Biomarkers Using Transcriptomic Data in Non-Small-Cell Lung Cancer, PLoS One. (2013) 8, no. 12, e82241, 10.1371/journal.pone.0082241, 2-s2.0-84893181929, 24367507. 24367507 PMC3867325

[35] Vasaikar S. V. , Straub P. , Wang J. , and Zhang B. , LinkedOmics: Analyzing Multi-Omics Data Within and Across 32 Cancer Types, Nucleic Acids Research. (2018) 46, no. D1, D956–D963, 10.1093/nar/gkx1090, 2-s2.0-85040936922. 29136207 PMC5753188

[36] Zhang C. , Guo C. , Li Y. , Liu K. , Zhao Q. , and Ouyang L. , Identification of Claudin-6 as a Molecular Biomarker in Pan-Cancer Through Multiple Omics Integrative Analysis, Frontiers in Cell and Development Biology. (2021) 9, 726656, 10.3389/fcell.2021.726656, 34409042. PMC8365468 34409042

[37] Szklarczyk D. , Morris J. H. , Cook H. , Kuhn M. , Wyder S. , Simonovic M. , Santos A. , Doncheva N. T. , Roth A. , Bork P. , Jensen L. J. , and von Mering C. , The STRING Database in 2017: Quality-Controlled Protein–Protein Association Networks, Made Broadly Accessible, Nucleic Acids Research. (2017) 45, no. D1, D362–D368, 10.1093/nar/gkw937, 2-s2.0-85021857514. 27924014 PMC5210637

[38] Raudvere U. , Kolberg L. , Kuzmin I. , Arak T. , Adler P. , Peterson H. , and Vilo J. , g:Profiler: A Web Server for Functional Enrichment Analysis and Conversions of Gene Lists (2019 Update), Nucleic Acids Research. (2019) 47, no. W1, W191–w198, 10.1093/nar/gkz369, 2-s2.0-85068995138, 31066453. 31066453 PMC6602461

[39] Excoffier L. , Controlling the False Discovery Rate: A Practical and Powerful Approach to Multiple Testing, Journal of the Royal Statistical Society: Series B. (1995) 57, 564–567.

[40] Howe K. L. , Contreras-Moreira B. , De Silva N. , Maslen G. , Akanni W. , Allen J. , Alvarez-Jarreta J. , Barba M. , Bolser D. M. , Cambell L. , Carbajo M. , Chakiachvili M. , Christensen M. , Cummins C. , Cuzick A. , Fexova S. , Gall A. , George N. , Gil L. , Gupta P. , Hammond-Kosack K. E. , Haskell E. , Hunt S. E. , Jaiswal P. , Janacek S. H. , Kersey P. J. , Langridge N. , Maheswari U. , Maurel T. , McDowall M. D. , Moore B. , Muffato M. , Naamati G. , Naithani S. , Olson A. , Papatheodorou I. , Patricio M. , Paulini M. , Pedro H. , Perry E. , Preece J. , Rosello M. , Russell M. , Sitnik V. , Staines D. M. , Stein J. , Tello-Ruiz M. K. , Trevanion S. J. , Urban M. , Wei S. , Ware D. , Williams G. , Yates A. D. , and Flicek P. , Ensembl Genomes 2020-Enabling Non-Vertebrate Genomic Research, Nucleic Acids Research. (2020) 48, no. D1, D689–d695. 31598706 10.1093/nar/gkz890 PMC6943047

[41] Ringnér M. , Fredlund E. , Häkkinen J. , Borg Å. , and Staaf J. , GOBO: Gene Expression-Based Outcome for Breast Cancer Online, PLoS One. (2011) 6, no. 3, e17911, 10.1371/journal.pone.0017911, 2-s2.0-79952819724, 21445301. 21445301 PMC3061871

[42] Tancoš V. and Blichárová A. , Predictive Biomarkers of Response to Immunotherapy in Triple-Negative Breast Cancer - State of the Art and Future Perspectives, Klinická Onkologie. (2023) 36, no. 1, 28–34, 10.48095/ccko202328, 36868830. 36868830

[43] Gajewski T. F. , Schreiber H. , and Fu Y. X. , Innate and Adaptive Immune Cells in the Tumor Microenvironment, Nature Immunology. (2013) 14, no. 10, 1014–1022, 10.1038/ni.2703, 2-s2.0-84886698315, 24048123. 24048123 PMC4118725

[44] Jones P. A. and Baylin S. B. , The Epigenomics of Cancer, Cell. (2007) 128, no. 4, 683–692, 10.1016/j.cell.2007.01.029, 2-s2.0-33847065486, 17320506. 17320506 PMC3894624

[45] Skvortsova K. , Stirzaker C. , and Taberlay P. , The DNA Methylation Landscape in Cancer, Essays in Biochemistry. (2019) 63, no. 6, 797–811, 10.1042/EBC20190037, 31845735. 31845735 PMC6923322

[46] Gamage S. M. K. , Nanayakkara S. , Macfarlane L. , Hewage D. , Cheng T. , Aktar S. , Lu C. T. , Dissabandara L. , Islam F. , Lam A. K. , and Gopalan V. , Heme Oxygenase-1 & 2 and Their Potential Contribution in Heme Induced Colorectal Carcinogenesis, Pathology, Research and Practice. (2022) 233, 153885, 10.1016/j.prp.2022.153885. 35428017

[47] Severance S. and Hamza I. , Trafficking of Heme and Porphyrins in Metazoa, Chemical Reviews. (2009) 109, no. 10, 4596–4616, 10.1021/cr9001116, 2-s2.0-70350686755, 19764719. 19764719 PMC2769250

[48] Porporato P. E. , Filigheddu N. , Pedro J. M. B. , Kroemer G. , and Galluzzi L. , Mitochondrial Metabolism and Cancer, Cell Research. (2018) 28, no. 3, 265–280, 10.1038/cr.2017.155, 2-s2.0-85042880682, 29219147. 29219147 PMC5835768

[49] Hossen M. A. , Reza M. S. , Rana M. M. , Hossen M. B. , Shoaib M. , Mollah M. N. H. , and Han C. , Identification of Most Representative Hub-Genes for Diagnosis, Prognosis, and Therapies of Hepatocellular Carcinoma, Chinese Clinical Oncology. (2024) 13, no. 3, 10.21037/cco-23-151, 38984486. 38984486

[50] Krukowska K. and Magierowski M. , Carbon Monoxide (CO)/Heme Oxygenase (HO)-1 in Gastrointestinal Tumors Pathophysiology and Pharmacology - Possible Anti- and Pro-Cancer Activities, Biochemical Pharmacology. (2022) 201, 115058, 10.1016/j.bcp.2022.115058, 35490732. 35490732

[51] Hassannia B. , Vandenabeele P. , and Vanden Berghe T. , Targeting Ferroptosis to Iron Out Cancer, Cancer Cell. (2019) 35, no. 6, 830–849, 10.1016/j.ccell.2019.04.002, 2-s2.0-85066481344. 31105042

[52] Ni C. , Zhao X. , Sun T. , Liu Y. , Gu Q. , and Sun B. , Role of Gastrin-Releasing Peptides in Breast Cancer Metastasis, Human Pathology. (2012) 43, no. 12, 2342–2347, 10.1016/j.humpath.2012.04.007, 2-s2.0-84869506790, 22789785. 22789785

