# Supplementary material for: HCCS Serves as Potential Prognostic Biomarker and Therapeutic Target in Human Breast Cancer
Source: Int J Breast Cancer. 2025 Dec 5;2025:6717594. doi: 10.1155/ijbc/6717594 (PMC12752879; doi:10.1155/ijbc/6717594)
Supplement: Supplementary file 1 — Supporting Information Additional supporting information can be found online in the Supporting Information section. Figure S1. Overview of HCCS gene expression in different human tissue samples. Figure S2. Differential HCCS expression in TCGA cancer data sets. Figure S3. HCCS expression with immune infiltration profile in breast cancer subtypes. Figure S4. Protein–protein interaction network of HCCS. [file IJBC-2025-6717594-s001.zip › Supporting information__IJBC.docx]

**Title: HCCS serves as potential prognostic biomarker and therapeutic target in human breast cancer**

**Sm Faysal Bellah^1, 2, *^, Md Alim Hossen^3^, S M Saker Billah^4^, and Md. Nur Islam^5^**

^1^Department of Pharmacy, Bangladesh University, Dhaka-1207, Bangladesh.

^2^ Bangladesh University Research Center, Bangladesh University, Dhaka-1207, Bangladesh.

^3^Bioinformatics Laboratory, Department of Statistics, University of Rajshahi, Rajshahi-6205, Bangladesh.

^4^Department of Chemistry, National University, Gazipur-1704, Bangladesh.

^5^Department of Pharmacy, Manarat International University, Dhaka-1341, Bangladesh

**Short Title: HCCS and Breast Cancer Risks.**

^*^Corresponding author:

**Dr. Sm Faysal Bellah**

**Associate Professor**

Department of Pharmacy

Bangladesh University

Dhaka-1207, Bangladesh

Tel: +8801844775670, +8801913261838

E-mail: faysal_phku@yahoo.com

Orchid ID: https://orcid.org/0000-0002-8626-8547


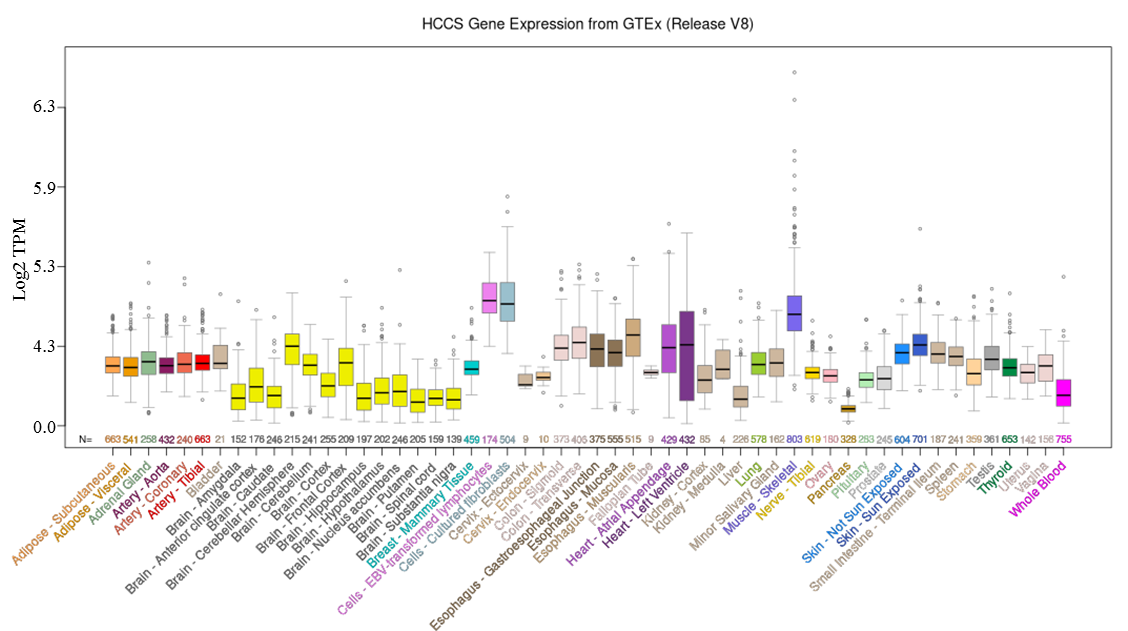


**Figure S1: Overview of HCCS gene expression in different human tissues sample**. UCSC obtained the gene-level expression files, gene annotations and sample metadata from the genotype-tissue expression (GTEx) portal. Median expression level were computed as log2-transcript per million (Log2-TPM) per gene/per tissue (<http://genome.ucsc.edu/cgi-bin/hgc?hgsid=2519955909_BqMGqVPlPINAgXQsWpOhGjNBQFa3&db=hg38&c=chrX&l=11111300&r=11123078&o=11111300&t=11123078&g=gtexGeneV8&i=HCCS>).


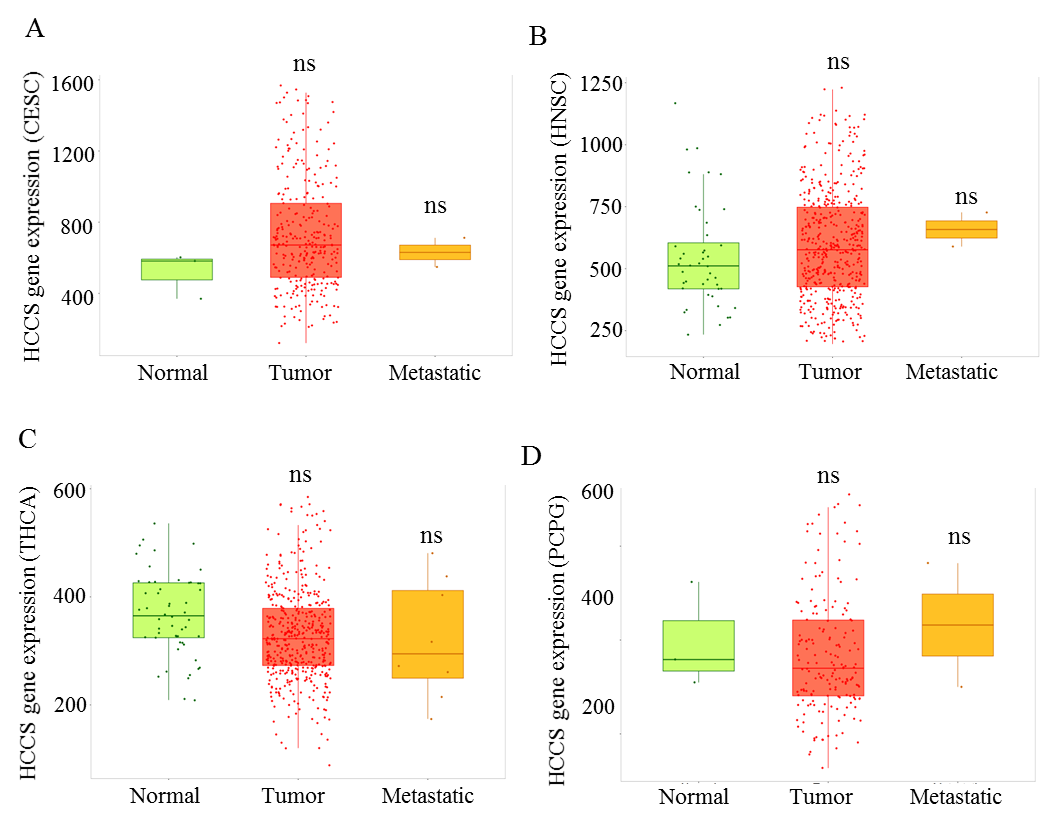


**Figure S2. Differential HCCS expression in TCGA cancer data sets.** HCCS gene expression in (A) cervical squamous cell carcinoma and endocervical adenocarcinoma (CESC) (B) head and neck squamous cell carcinoma (HNSC) (C) thyroid carcinoma (THCA) (D) pheochromocytoma and paraganglioma (PCPG) accounting the normal, tumor, and metastatic using RNA-Seq based data (<https://tnmplot.com/analysis/>). Statistical significance was determined using the Dunnet test, and the p value were calculated as compared to that of normal. The significance levels indicated as follows: ns: not significant, *p < 0.05, **p < 0.01, and ***p < 0.001.


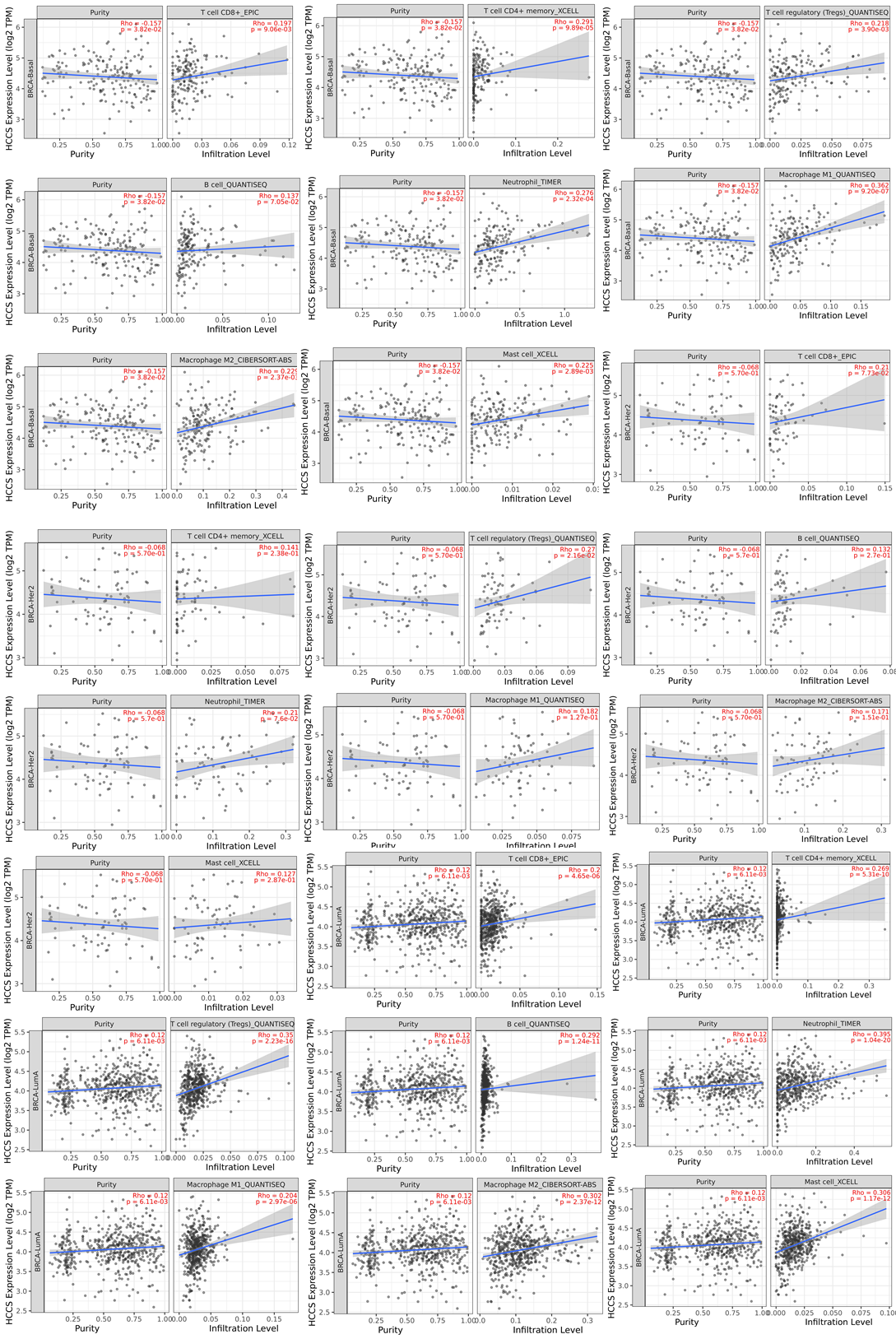


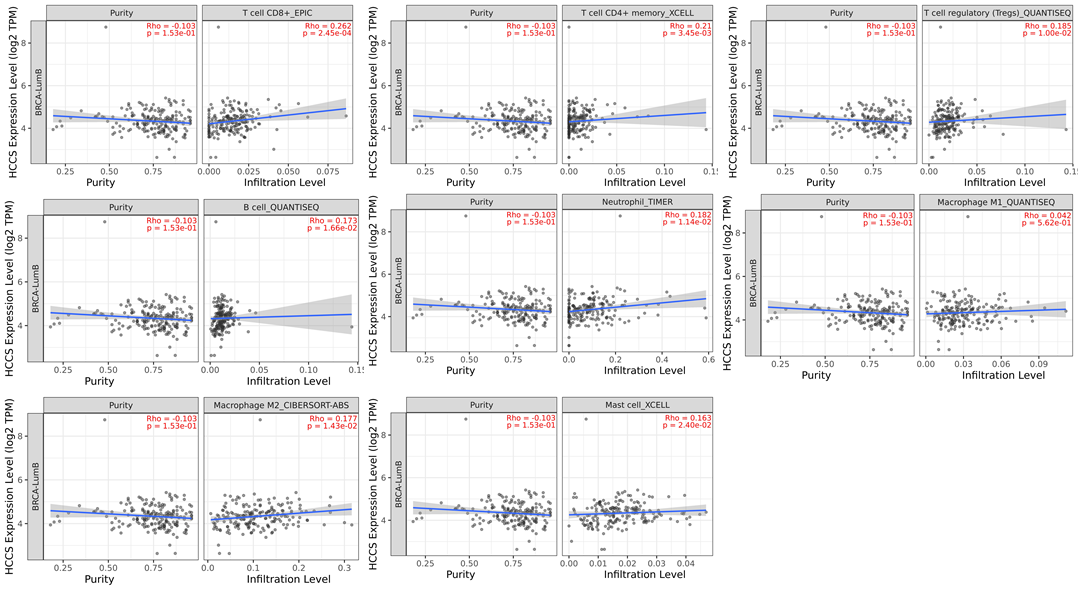


**Figure S3:** **HCCS expression with immune infiltration profile in breast cancer sub-types.** Scatter plots illustrated the Spearman correlation between HCCS expression and various immune cell types, including CD8+ T cells, CD4+ T cells, regulatory T cells, memory B cells, neutrophils, M1 macrophages, M2 macrophages, and mast cells. These correlations varied across different breast cancer subtypes—namely, basal-like, HER2-enriched, luminal A, and luminal B—based on transcriptomic data from breast cancer patients. The *p* values < 0.05 were considered statistically significant using the TCGA breast cancer data cohort.


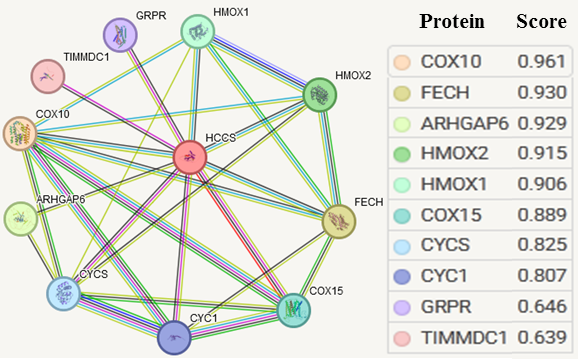


**Figure S4: Protein–protein interaction network of HCCS**. The left panel presents a schematic representation of the predicted interaction network involving HCCS and its associated binding partners. The right panel provides detailed information on each interacting protein, including interaction confidence scores derived from STRING analysis (<https://cn.string-b.org/cgi/network>).

**Table S1:** The significant enrichments of HCCS correlated genes in GO annotation for MFs, BPs, CCs, and KEGG Source.

| **Source** | **Term_Name** | **Term_ID** | **Padj.Value** | **Enriched Genes** |
| --- | --- | --- | --- | --- |
| GO:MF | heme binding | GO:0020037 | 1.82E-11 | HCCS, FECH, HMOX2, HMOX1, COX15, CYCS, CYC1 |
| GO:MF | tetrapyrrole binding | GO:0046906 | 1.82E-11 | HCCS, FECH, HMOX2, HMOX1, COX15, CYCS, CYC1 |
| GO:MF | heme oxygenase (decyclizing) activity | GO:0004392 | 7.87E-06 | HMOX2, HMOX1 |
| GO:MF | oxidoreductase activity, acting on paired donors, with incorporation or reduction of molecular oxygen, reduced flavin or flavoprotein as one donor, and incorporation of one atom of oxygen | GO:0016712 | 5.98E-03 | HMOX2, HMOX1 |
| GO:MF | oxidoreductase activity | GO:0016491 | 5.98E-03 | HMOX2, HMOX1, COX15, CYC1 |
| GO:MF | protoheme IX farnesyltransferase activity | GO:0008495 | 5.98E-03 | COX10 |
| GO:MF | holocytochrome-c synthase activity | GO:0004408 | 5.98E-03 | HCCS |
| GO:MF | ferrochelatase activity | GO:0004325 | 5.98E-03 | FECH |
| GO:MF | iron-responsive element binding | GO:0030350 | 1.23E-02 | FECH |
| GO:MF | geranylgeranyl diphosphate synthase activity | GO:0004311 | 1.23E-02 | COX10 |
| GO:MF | monooxygenase activity | GO:0004497 | 1.23E-02 | HMOX2, HMOX1 |
| GO:MF | electron transfer activity | GO:0009055 | 1.23E-02 | CYCS, CYC1 |
| GO:MF | ubiquinol-cytochrome-c reductase activity | GO:0008121 | 2.05E-02 | CYC1 |
| GO:MF | metal ion binding | GO:0046872 | 2.05E-02 | HCCS, FECH, HMOX2, HMOX1, COX15, CYCS, CYC1 |
| GO:MF | oxidoreductase activity, acting on diphenols and related substances as donors | GO:0016679 | 2.14E-02 | CYC1 |
| GO:MF | prenyl diphosphate synthase activity | GO:0120531 | 2.14E-02 | COX10 |
| GO:MF | oxidoreductase activity, acting on paired donors, with incorporation or reduction of molecular oxygen | GO:0016705 | 2.14E-02 | HMOX2, HMOX1 |
| GO:MF | cation binding | GO:0043169 | 2.14E-02 | HCCS, FECH, HMOX2, HMOX1, COX15, CYCS, CYC1 |
| GO:MF | lyase activity | GO:0016829 | 2.86E-02 | HCCS, FECH |
| GO:MF | oxidoreductase activity, acting on NAD(P)H, heme protein as acceptor | GO:0016653 | 2.86E-02 | COX15 |
| GO:MF | carbon-sulfur lyase activity | GO:0016846 | 3.40E-02 | HCCS |
| GO:MF | catalytic activity | GO:0003824 | 3.77E-02 | HCCS, COX10, FECH, ARHGAP6, HMOX2, HMOX1, COX15, CYC1 |
| GO:MF | prenyltransferase activity | GO:0004659 | 3.93E-02 | COX10 |
| GO:MF | phospholipase activator activity | GO:0016004 | 3.97E-02 | ARHGAP6 |
| GO:MF | phospholipase binding | GO:0043274 | 4.37E-02 | ARHGAP6 |
| GO:MF | lipase activator activity | GO:0060229 | 4.39E-02 | ARHGAP6 |
| GO:MF | ferrous iron binding | GO:0008198 | 4.40E-02 | FECH |
| GO:BP | heme metabolic process | GO:0042168 | 5.85E-09 | COX10, FECH, HMOX2, HMOX1, COX15 |
| GO:BP | porphyrin-containing compound metabolic process | GO:0006778 | 5.93E-09 | COX10, FECH, HMOX2, HMOX1, COX15 |
| GO:BP | tetrapyrrole metabolic process | GO:0033013 | 9.37E-09 | COX10, FECH, HMOX2, HMOX1, COX15 |
| GO:BP | pigment metabolic process | GO:0042440 | 2.31E-08 | COX10, FECH, HMOX2, HMOX1, COX15 |
| GO:BP | heme A biosynthetic process | GO:0006784 | 9.95E-07 | COX10, FECH, COX15 |
| GO:BP | heme a metabolic process | GO:0046160 | 9.95E-07 | COX10, FECH, COX15 |
| GO:BP | heme oxidation | GO:0006788 | 1.36E-05 | HMOX2, HMOX1 |
| GO:BP | heme biosynthetic process | GO:0006783 | 2.23E-05 | COX10, FECH, COX15 |
| GO:BP | tetrapyrrole biosynthetic process | GO:0033014 | 2.57E-05 | COX10, FECH, COX15 |
| GO:BP | porphyrin-containing compound biosynthetic process | GO:0006779 | 2.57E-05 | COX10, FECH, COX15 |
| GO:BP | cytochrome complex assembly | GO:0017004 | 3.75E-05 | HCCS, COX10, COX15 |
| GO:BP | generation of precursor metabolites and energy | GO:0006091 | 5.32E-05 | HCCS, COX10, FECH, CYCS, CYC1 |
| GO:BP | pigment biosynthetic process | GO:0046148 | 8.49E-05 | COX10, FECH, COX15 |
| GO:BP | cellular respiration | GO:0045333 | 1.14E-04 | HCCS, COX10, CYCS, CYC1 |
| GO:BP | heme O metabolic process | GO:0048033 | 2.14E-04 | COX10, FECH |
| GO:BP | heme O biosynthetic process | GO:0048034 | 2.14E-04 | COX10, FECH |
| GO:BP | heme catabolic process | GO:0042167 | 2.90E-04 | HMOX2, HMOX1 |
| GO:BP | pigment catabolic process | GO:0046149 | 2.90E-04 | HMOX2, HMOX1 |
| GO:BP | tetrapyrrole catabolic process | GO:0033015 | 3.37E-04 | HMOX2, HMOX1 |
| GO:BP | mitochondrial electron transport, ubiquinol to cytochrome c | GO:0006122 | 3.37E-04 | CYCS, CYC1 |
| GO:BP | porphyrin-containing compound catabolic process | GO:0006787 | 3.37E-04 | HMOX2, HMOX1 |
| GO:BP | energy derivation by oxidation of organic compounds | GO:0015980 | 3.37E-04 | HCCS, COX10, CYCS, CYC1 |
| GO:BP | respiratory electron transport chain | GO:0022904 | 3.78E-04 | HCCS, CYCS, CYC1 |
| GO:BP | electron transport chain | GO:0022900 | 4.77E-04 | HCCS, CYCS, CYC1 |
| GO:BP | multicellular organismal-level iron ion homeostasis | GO:0060586 | 1.43E-03 | FECH, HMOX1 |
| GO:BP | aerobic respiration | GO:0009060 | 1.65E-03 | COX10, CYCS, CYC1 |
| GO:BP | response to arsenic-containing substance | GO:0046685 | 1.65E-03 | FECH, HMOX1 |
| GO:BP | protein-heme linkage | GO:0017003 | 7.42E-03 | HCCS |
| GO:BP | protein-tetrapyrrole linkage | GO:0017006 | 7.42E-03 | HCCS |
| GO:BP | cytochrome c-heme linkage | GO:0018063 | 7.42E-03 | HCCS |
| GO:BP | regulation of plasma lipoprotein particle levels | GO:0097006 | 8.37E-03 | FECH, HMOX1 |
| GO:BP | multicellular organismal-level chemical homeostasis | GO:0140962 | 9.22E-03 | FECH, HMOX1 |
| GO:BP | aerobic electron transport chain | GO:0019646 | 1.16E-02 | CYCS, CYC1 |
| GO:BP | regulation of eIF2 alpha phosphorylation by heme | GO:0010999 | 1.16E-02 | FECH |
| GO:BP | mitochondrial ATP synthesis coupled electron transport | GO:0042775 | 1.16E-02 | CYCS, CYC1 |
| GO:BP | ATP synthesis coupled electron transport | GO:0042773 | 1.16E-02 | CYCS, CYC1 |
| GO:BP | smooth muscle hyperplasia | GO:0014806 | 1.16E-02 | HMOX1 |
| GO:BP | detection of UV | GO:0009589 | 1.16E-02 | FECH |
| GO:BP | muscle hyperplasia | GO:0014900 | 1.16E-02 | HMOX1 |
| GO:BP | cellular response to cisplatin | GO:0072719 | 1.63E-02 | HMOX1 |
| GO:BP | response to platinum ion | GO:0070541 | 1.63E-02 | FECH |
| GO:BP | positive regulation of respiratory gaseous exchange | GO:1903942 | 2.02E-02 | GRPR |
| GO:BP | response to cisplatin | GO:0072718 | 2.02E-02 | HMOX1 |
| GO:BP | smooth muscle adaptation | GO:0014805 | 2.02E-02 | HMOX1 |
| GO:BP | oxidative phosphorylation | GO:0006119 | 2.14E-02 | CYCS, CYC1 |
| GO:BP | erythrocyte homeostasis | GO:0034101 | 2.14E-02 | FECH, HMOX1 |
| GO:BP | response to methylmercury | GO:0051597 | 2.32E-02 | FECH |
| GO:BP | psychomotor behavior | GO:0036343 | 2.32E-02 | GRPR |
| GO:BP | response to insecticide | GO:0017085 | 2.72E-02 | FECH |
| GO:BP | myeloid cell homeostasis | GO:0002262 | 2.97E-02 | FECH, HMOX1 |
| GO:BP | positive regulation of behavioral fear response | GO:2000987 | 3.05E-02 | GRPR |
| GO:BP | positive regulation of fear response | GO:1903367 | 3.23E-02 | GRPR |
| GO:BP | regulation of hemoglobin biosynthetic process | GO:0046984 | 3.23E-02 | FECH |
| GO:BP | heme B metabolic process | GO:0046492 | 3.23E-02 | FECH |
| GO:BP | heme B biosynthetic process | GO:0006785 | 3.23E-02 | FECH |
| GO:BP | negative regulation of ferroptosis | GO:0110076 | 3.39E-02 | HMOX1 |
| GO:BP | regulation of translational initiation by eIF2 alpha phosphorylation | GO:0010998 | 3.39E-02 | FECH |
| GO:BP | wound healing involved in inflammatory response | GO:0002246 | 3.39E-02 | HMOX1 |
| GO:BP | positive regulation of blood vessel endothelial cell proliferation involved in sprouting angiogenesis | GO:1903589 | 3.39E-02 | HMOX1 |
| GO:BP | regulation of behavioral fear response | GO:2000822 | 4.07E-02 | GRPR |
| GO:BP | very-low-density lipoprotein particle assembly | GO:0034379 | 4.10E-02 | FECH |
| GO:BP | regulation of fear response | GO:1903365 | 4.10E-02 | GRPR |
| GO:BP | regulation of ferroptosis | GO:0110075 | 4.10E-02 | HMOX1 |
| GO:BP | protoporphyrinogen IX metabolic process | GO:0046501 | 4.10E-02 | FECH |
| GO:BP | positive regulation of phospholipase activity | GO:0010518 | 4.10E-02 | ARHGAP6 |
| GO:BP | ferroptosis | GO:0097707 | 4.31E-02 | HMOX1 |
| GO:BP | regulation of translational initiation in response to stress | GO:0043558 | 4.31E-02 | FECH |
| GO:BP | hemoglobin biosynthetic process | GO:0042541 | 4.57E-02 | FECH |
| GO:CC | mitochondrial envelope | GO:0005740 | 2.19E-08 | HCCS, COX10, FECH, HMOX1, COX15, CYCS, CYC1, TIMMDC1 |
| GO:CC | mitochondrial membrane | GO:0031966 | 2.19E-08 | HCCS, COX10, FECH, HMOX1, COX15, CYCS, CYC1, TIMMDC1 |
| GO:CC | mitochondrial inner membrane | GO:0005743 | 2.51E-08 | HCCS, COX10, FECH, COX15, CYCS, CYC1, TIMMDC1 |
| GO:CC | organelle inner membrane | GO:0019866 | 3.91E-08 | HCCS, COX10, FECH, COX15, CYCS, CYC1, TIMMDC1 |
| GO:CC | organelle envelope | GO:0031967 | 3.28E-07 | HCCS, COX10, FECH, HMOX1, COX15, CYCS, CYC1, TIMMDC1 |
| GO:CC | mitochondrion | GO:0005739 | 3.53E-06 | HCCS, COX10, FECH, HMOX1, COX15, CYCS, CYC1, TIMMDC1 |
| GO:CC | cytochrome complex | GO:0070069 | 4.55E-06 | COX10, COX15, CYC1 |
| GO:CC | organelle membrane | GO:0031090 | 4.91E-05 | HCCS, COX10, FECH, HMOX2, HMOX1, COX15, CYCS, CYC1, TIMMDC1 |
| GO:CC | respiratory chain complex | GO:0098803 | 8.87E-03 | COX15, CYC1 |
| GO:CC | apoptosome | GO:0043293 | 1.06E-02 | CYCS |
| GO:CC | respiratory chain complex III | GO:0045275 | 3.22E-02 | CYC1 |
| KEGG | Porphyrin metabolism | KEGG:00860 | 4.80E-11 | HCCS, COX10, FECH, HMOX2, HMOX1, COX15 |
| KEGG | Metabolic pathways | KEGG:01100 | 1.41E-04 | HCCS, COX10, FECH, HMOX2, HMOX1, COX15, CYCS, CYC1 |
| KEGG | Oxidative phosphorylation | KEGG:00190 | 1.41E-04 | COX10, COX15, CYCS, CYC1 |
| KEGG | Biosynthesis of cofactors | KEGG:01240 | 5.46E-03 | COX10, FECH, COX15 |
| KEGG | Mineral absorption | KEGG:04978 | 1.40E-02 | HMOX2, HMOX1 |
| KEGG | Thermogenesis | KEGG:04714 | 1.40E-02 | COX10, COX15, CYC1 |
