# Supplementary material for: HCCS Serves as Potential Prognostic Biomarker and Therapeutic Target in Human Breast Cancer
Source: Int J Breast Cancer. 2025 Dec 5;2025:6717594. doi: 10.1155/ijbc/6717594 (PMC12752879; doi:10.1155/ijbc/6717594)
Supplement: Supplementary file 1 — Supporting Information Additional supporting information can be found online in the Supporting Information section. Figure S1. Overview of HCCS gene expression in different human tissue samples. Figure S2. Differential HCCS expression in TCGA cancer data sets. Figure S3. HCCS expression with immune infiltration profile in breast cancer subtypes. Figure S4. Protein–protein interaction network of HCCS. [file IJBC-2025-6717594-s001.zip › HCCS_Figures_IJBC_Log2_Supplemenatary.pptx]

## Slide 1
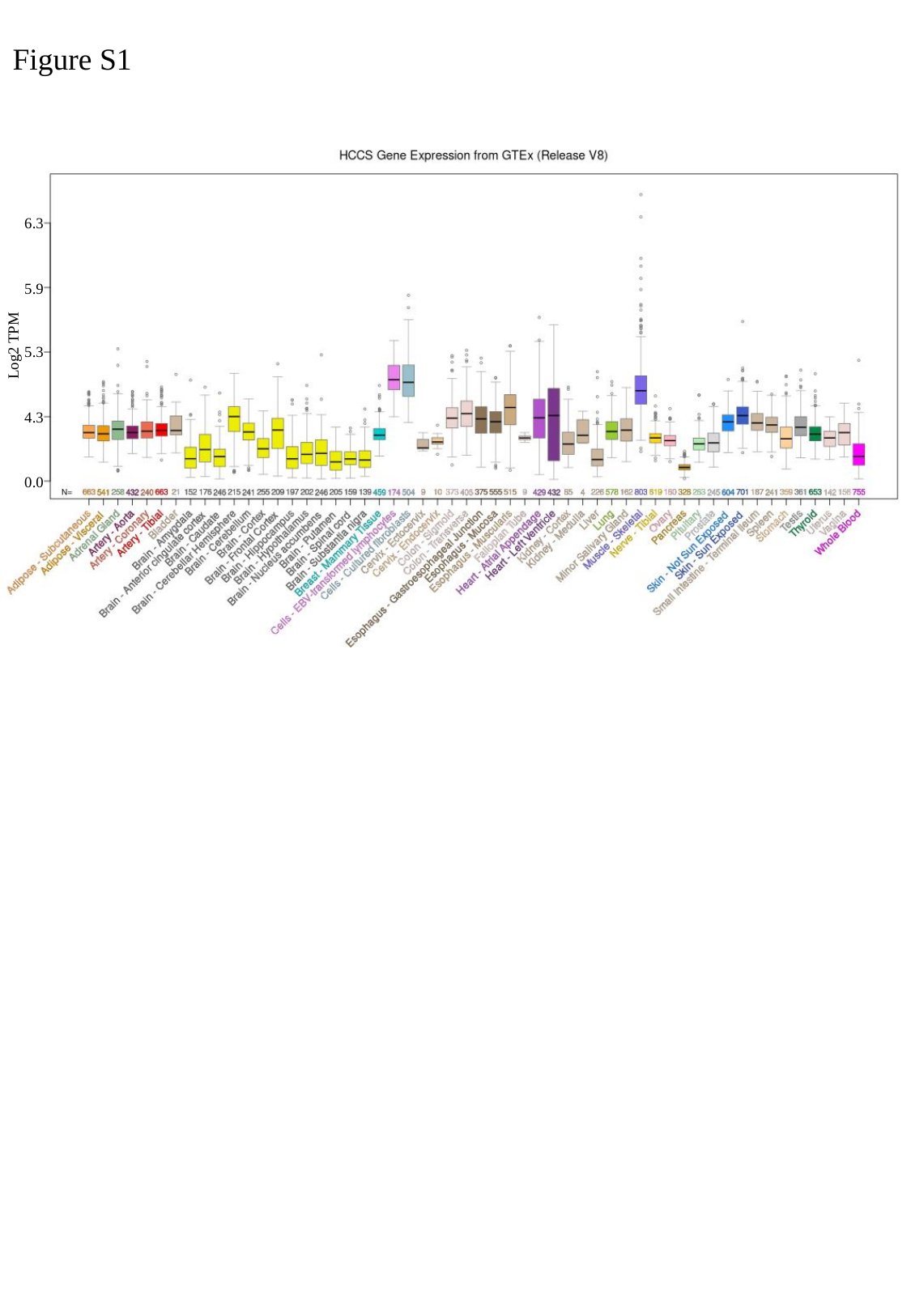

Figure S1
6.3
5.9
Log2 TPM
5.3
4.3
0.0

## Slide 2
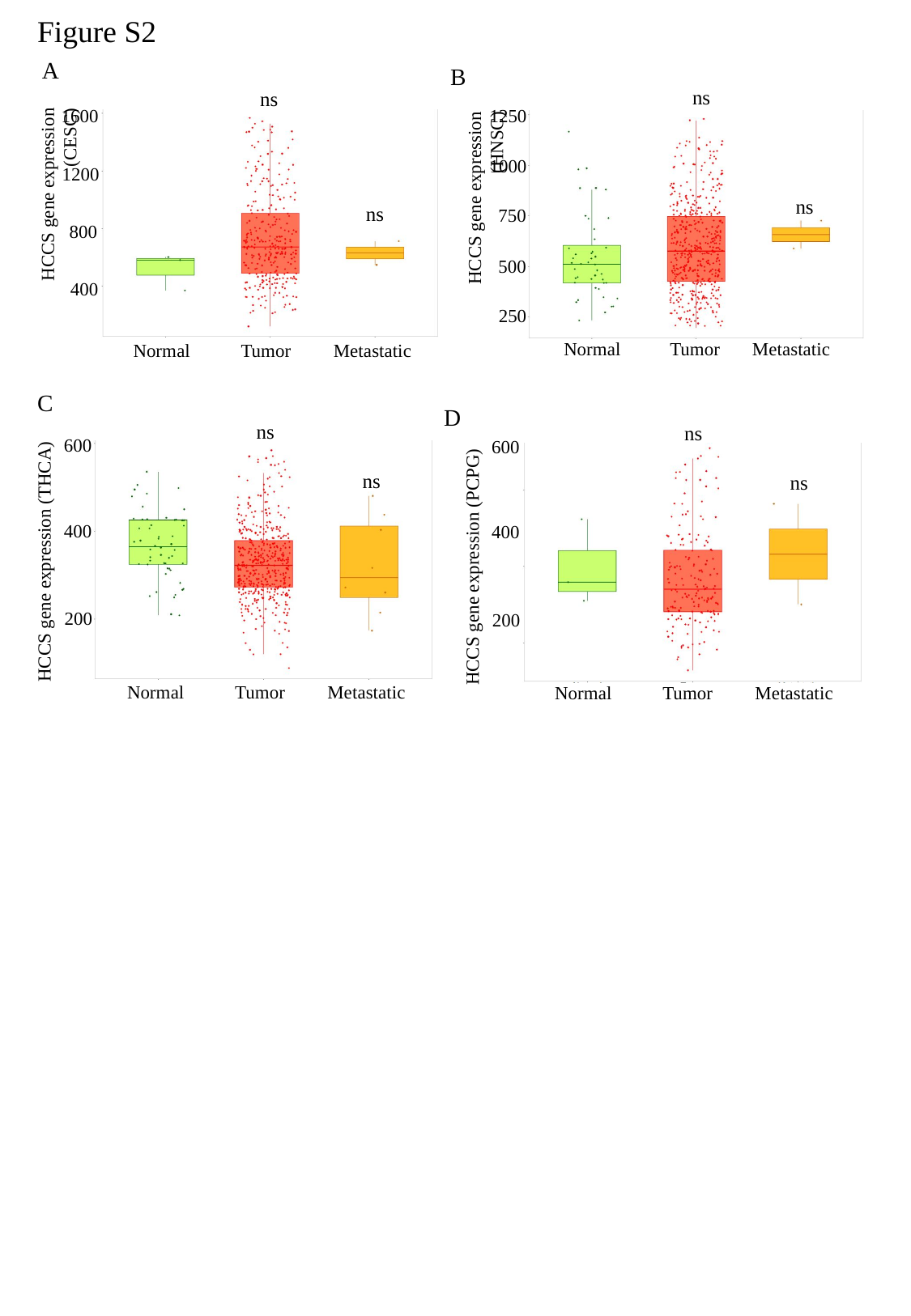

Figure S2
A
B
ns
ns
1600
1250
1000
1200
ns
ns
750
HCCS gene expression (CESC)
HCCS gene expression (HNSC)
800
500
400
250
Normal
Tumor
Metastatic
Normal
Tumor
Metastatic
C
D
ns
ns
600
600
ns
ns
400
400
HCCS gene expression (PCPG)
HCCS gene expression (THCA)
200
200
Normal
Tumor
Metastatic
Normal
Tumor
Metastatic

## Slide 3
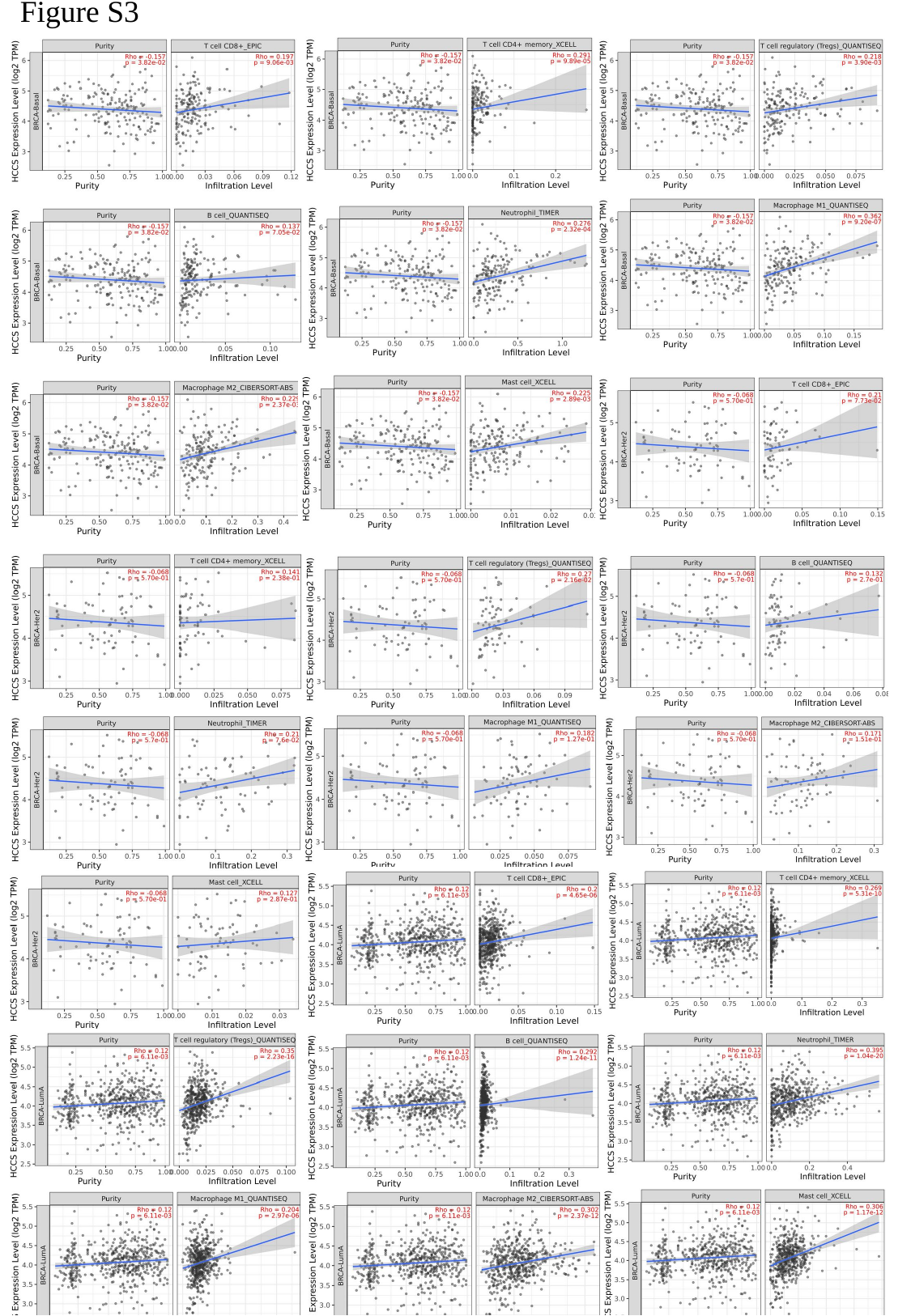

Figure S3

## Slide 4
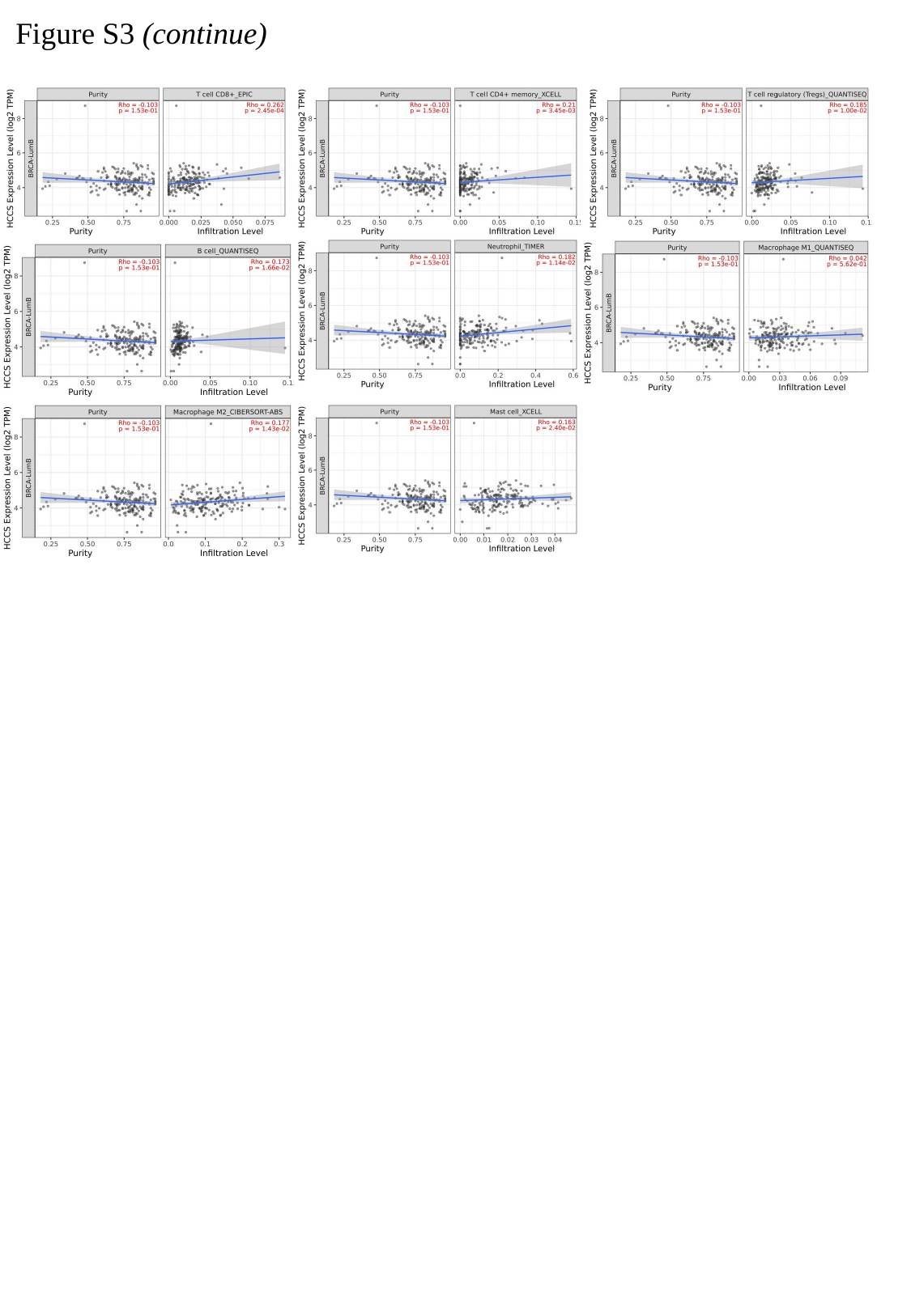

Figure S3 (continue)

## Slide 5
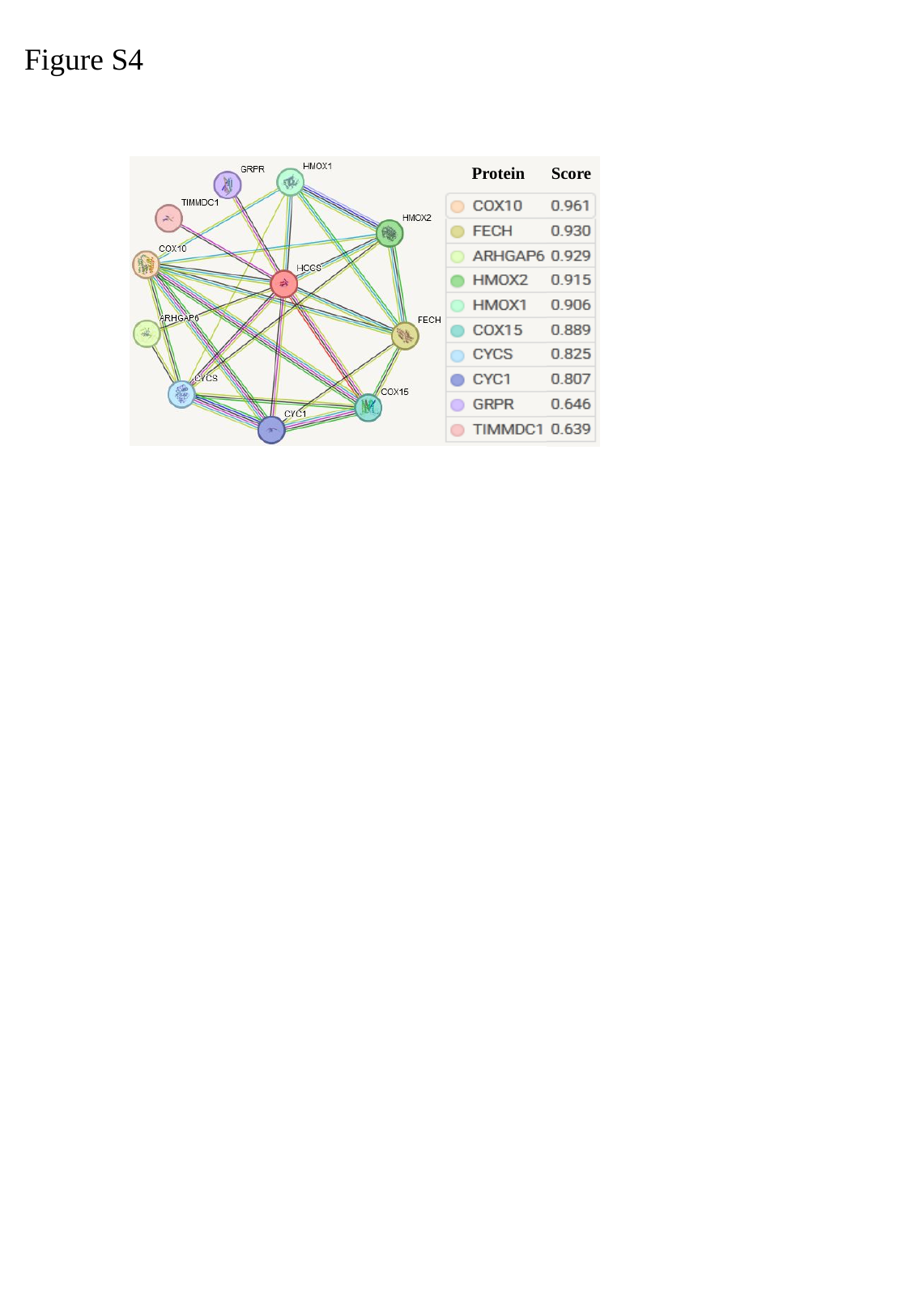

Figure S4
Protein
Score
